# De novo mapping of α-helix recognition sites on protein surfaces using unbiased libraries

**DOI:** 10.1073/pnas.2210435119

**Published:** 2022-12-19

**Authors:** Kunhua Li, Olena S. Tokareva, Ty M. Thomson, Sebastian C. T. Wahl, Tara L. Travaline, Jessica D. Ramirez, Santosh K. Choudary, Sorabh Agarwal, Ward G. Walkup, Tivoli J. Olsen, Matthew J. Brennan, Gregory L. Verdine, John H. McGee

**Affiliations:** ^a^FOG Pharmaceuticals Inc., Cambridge, MA 02140; ^b^Department of Stem Cell and Regenerative Biology, Harvard University, Cambridge, MA 02138; ^c^Department of Chemistry and Chemical Biology, Harvard University, Cambridge, MA 02138; ^d^Department of Molecular and Cellular Biology, Harvard University, Cambridge, MA 02138

**Keywords:** stapled peptides, phage display, protein structure, drug discovery, chemical biology

## Abstract

The α-helix is one of the most common protein surface recognition motifs found in nature, and its unique amide-cloaking properties also enable α-helical polypeptide motifs to exist in membranes. Together, these properties have inspired the development of α-helically constrained (Helicon) therapeutics that can enter cells and bind targets that have been considered “undruggable”, such as protein–protein interactions. To date, no general method for discovering α-helical binders to proteins has been reported, limiting Helicon drug discovery to only those proteins with previously characterized α-helix recognition sites, and restricting the starting chemical matter to those known α-helical binders. Here, we report a general and rapid screening method to empirically map the α-helix binding sites on a broad range of target proteins in parallel using large, unbiased Helicon phage display libraries and next-generation sequencing. We apply this method to screen six structurally diverse protein domains, only one of which had been previously reported to bind isolated α-helical peptides, discovering 20 families that collectively comprise several hundred individual Helicons. Analysis of 14 X-ray cocrystal structures reveals at least nine distinct α-helix recognition sites across these six proteins, and biochemical and biophysical studies show that these Helicons can block protein–protein interactions, inhibit enzymatic activity, induce conformational rearrangements, and cause protein dimerization. We anticipate that this method will prove broadly useful for the study of protein recognition and for the development of both biochemical tools and therapeutics for traditionally challenging protein targets.

## Significance

To target disease-causing proteins that are inaccessible to small-molecule or antibody therapies, we are exploring the use of a nature-inspired drug modality that exploits the intrinsically favorable properties of synthetically constrained α-helical polypeptides (Helicons). Here, we report a screening method that enables the de novo discovery of Helicons for protein targets without prior information on their α-helix binding properties, which has significantly limited the proteins and diseases for which Helicons could be discovered. We applied this method to six structurally diverse protein domains and found Helicons that block relevant protein–protein interactions, inhibit enzymatic activity, induce conformational rearrangements, and dimerize their targets, highlighting the strength of the approach in interrogating targets that have been considered “undruggable”.

Recent advances in identifying human disease targets have not been matched by advances in the ability to drug these targets. This actionability gap is largely due to the fact that neither of the two main classes of approved therapeutics – biologics and small molecules – can simultaneously address target accessibility and selective target engagement. Biologics, despite an impressive ability to engage diverse target proteins, are largely restricted to an extracellular operating theater, as their size and polarity render them unable to cross biological membranes. Small molecules, in contrast, can access the intracellular space, but cannot bind with high affinity and specificity to the vast majority of proteins that are found there (1).

This disconnect between the ability to identify disease targets and the ability to drug them with high strength and specificity has created an impetus to develop new classes of drugs – ones that can engage intracellular proteins that lack the deep hydrophobic pocket ordinarily required for small-molecule binding. In nature, such “undruggable” proteins are often targeted with macrocyclic molecules, frequently peptidic in structure, whose large size compared with small molecules enables them to bind with high affinity and specificity to protein surfaces.

Significant efforts have been made to elucidate the mechanisms of cell entry for these natural products, which possess molecular weights of 700 to 1,200 Da or higher, well beyond the typical range for cell penetration in small-molecule drug discovery (2). While the mechanisms of cell entry are complex and vary from molecule to molecule, a substantial body of research on peptidic macrocycles has highlighted the importance of desolvating amide protons and reducing their exposure to the membrane interior as a key driver in passive, thermal diffusion across the lipid bilayer (2, 3) – a phenomenon we refer to as amide-proton cloaking. The amide proton, present between every residue in a polypeptide chain, is highly electropositive and forms a strong hydrogen-bonding interaction with water. This poses a substantial hurdle for membrane permeability, since tightly bound solvent water molecules must be shed prior to entering the lipid bilayer. Exposed amide groups incur a further energetic penalty upon membrane entry due to unfavorable electrostatic interactions with the low-dielectric environment of the membrane interior. Consequently, most peptides and proteins are unable to cross membranes.

For peptide macrocycles that are able to permeate the membrane, these problematic amide protons are typically removed either by replacing the amide with an ester, replacing it with a methyl group, or cloaking it from solvent water through the formation of intramolecular hydrogen bonds between the amide proton groups and a hydrogen bond-accepting group elsewhere in the molecule, often a carbonyl. Indeed, the paradigmatic example of a natural peptide macrocycle that exhibits robust cytosolic exposure, cyclosporine A (CsA), employs both N-methylation and cloaking through transannular hydrogen bonding (4). Extensive work by several research groups has shown that these strategies can be applied as design principles to endow artificial macrocycles with the ability to cross membranes (5–7).

In the context of folded proteins, nature has offered an alternative structural solution to the problem of amide proton cloaking: the α-helix, a protein secondary structure that is defined by repeating intramolecular hydrogen bonds between the amide proton group of one residue and the carbonyl of the amino acid located four residues N terminal to it. The intrinsic ability of α-helices to cloak their own amide protons explains their widespread prevalence in natural transmembrane proteins (8). Nuclear-encoded transmembrane proteins in eukaryotes are almost exclusively α-helical, and the only alternative transmembrane fold found in nature is the bacterially derived β-barrel, a helical structure that also cloaks amide protons via an intramolecular hydrogen bonding network, albeit in a significantly larger structure than single α-helices that is impractical for the development of synthetic drugs.

Just as CsA has served as the inspiration for the design of mimetic head-to-tail cyclized peptide ligands, so have proteinaceous α-helices inspired efforts to recapitulate nature’s design features in small, synthetic, α-helically constrained peptides (Helicons) that are hyperstabilized through the incorporation of a structural brace, also known as a “staple” (9–12). One of these, the all-hydrocarbon staple formed by ring-closing metathesis, has been extensively studied and is the basis for a drug candidate that targets the challenging proteins MDM2 and MDMX, currently undergoing Phase II clinical trials (13, 14).

Rational design of Helicons is difficult given the inability to systematically define the α-helix binding sites on a protein’s surface, and to identify Helicons that bind to those sites. This limitation has restricted research on Helicons to only those protein targets for which naturally occurring or previously characterized α-helical binders were known, with the Helicons generated from fragments of the known binders (3). Here, we report a rapid, high-throughput screening platform utilizing phage display that enables an unbiased mapping of the α-helical interactome of a given protein without any prior knowledge of its structure or known binding partners. We show that this platform is capable of identifying α-helix binding sites on the surfaces of a range of protein folds, including many for which no α-helical binders are known to exist. Helicons that bind these sites are able to impact diverse protein functions, including inhibiting protein–protein interactions, inhibiting enzymatic activity, inducing dimerization, and inducing conformational changes. Analysis of 14 high-resolution crystal structures of Helicon–protein complexes across six different protein domains reveals a range of binding modes, all of which are “side-on”, i.e., mediated exclusively by Helicon side-chains rather than involving main chain amide interactions. This screening platform significantly expands the universe of proteins that can be bound by Helicons, and furthers the pursuit of targeting undruggable proteins.

## Results

### An α-Helical Stapling System Compatible with Phage Display.

**Fig. 1.**
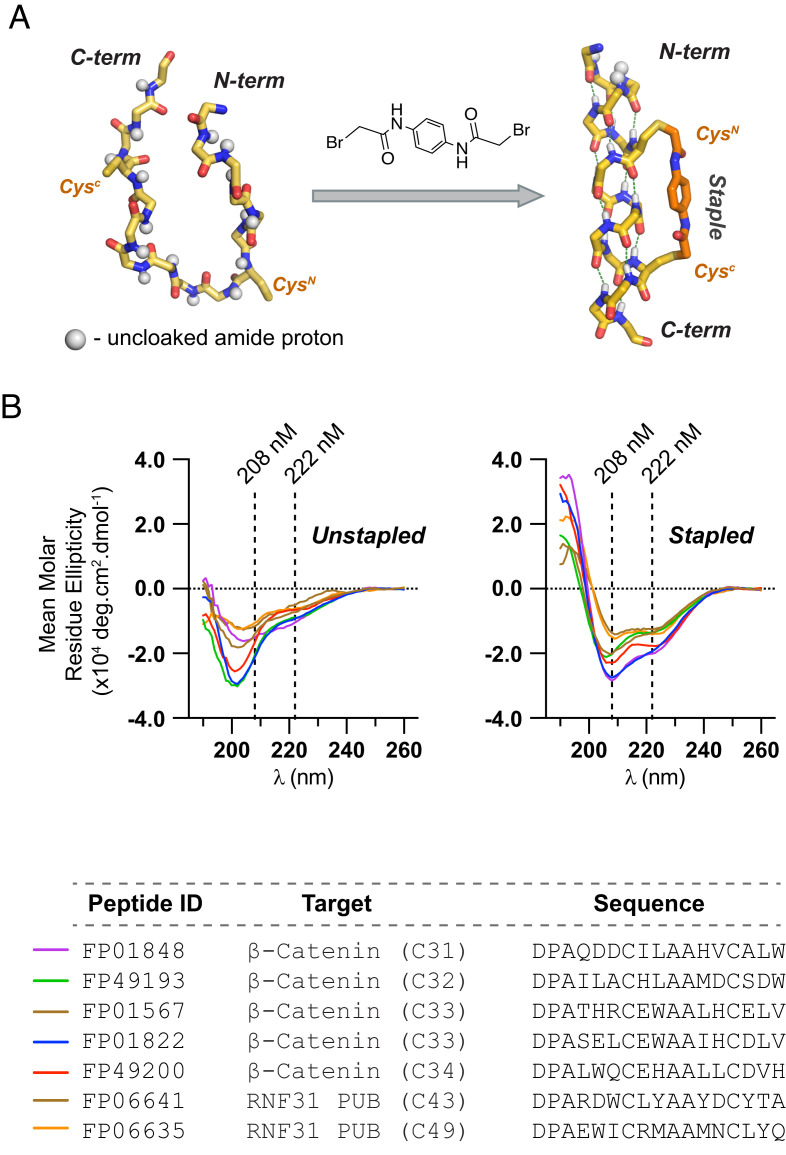
A cysteine stapling system that stabilizes Helicons in an α-helical conformation. (*A*) A bifunctional bromoacetamide cross-linker that staples two cysteine residues at *i, i+7* spacing. (*B*) Cysteine stapling increases the α-helical conformation in a panel of diverse Helicon sequences as assessed by circular dichroism spectropolarimetry.

We expected that an unbiased screening platform for identifying Helicon binders would necessitate very large library sizes to be successful with most targets. Of the ultra-high-throughput screening formats that have been established for peptides, we selected phage display, due to its combination of high library diversities, tolerance to chemical manipulation, and rapid and inexpensive library generation (15–17). We first wished to identify an appropriate reagent for α-helical stapling that would be compatible with large-scale modification of peptides presented by phage particles, and chose to focus on cross-linking cysteine residues with alkyl bromides, which can be performed under aqueous conditions and in the presence of other biomacromolecules (18). Peptide model studies evaluating a panel of bifunctional alkyl bromides identified N, N′-(1,4-phenylene)bis(2-bromoacetamide) as a promising reagent for cross-linking pairs of cysteines spaced two turns apart on the same helix face at *i, i+7* positioning (Fig. 1*A* and *SI Appendix*, Fig. S1). Incorporating this staple into a diverse panel of seven 14-amino acid peptides from this work consistently led to an increase in α-helical secondary structure, as assessed by circular dichroism spectropolarimetry and quantitation of those data (Fig. 1*B*). In most cases, helicity was improved more than twofold (*SI Appendix*, Table S1), as expected based on other stapling systems (9).

To demonstrate that this chemistry could be performed directly on phage-displayed Helicons, we prepared M13 bacteriophage with a model sequence consisting of a pair of cysteine residues at positions *i* and *i+7* (*i, i+7)* fused to the pIII coat protein. Cysteine cross-linking on the phage surface has previously been demonstrated, but it is not trivial due to the presence of multiple structural disulfide bonds in the phage pIII protein, and the fact that phage-displayed polypeptides with pairs of cysteines are initially present in the phage particle in the oxidized, disulfide-bonded state (19–21). After comprehensive optimization of timing and the concentrations of phage, reducing agent, and cross-linker, we found that on-phage cross-linking could be achieved by briefly treating the phage with dithiothreitol, followed by rapid dialysis, incubation with N, N′-(1,4-phenylene)bis(2-bromoacetamide), and a final capping and quenching step (see *Methods*). We confirmed the presence of properly cross-linked Helicon by trypsinizing the phage and analyzing them by mass spectrometry (MS). Phage prepared in this way remained viable and compatible with downstream screening and sequencing (*SI Appendix*, Fig. S2*A* and below).

### Highly Parallel Screening of Helicon Phage Libraries.

**Fig. 2.**
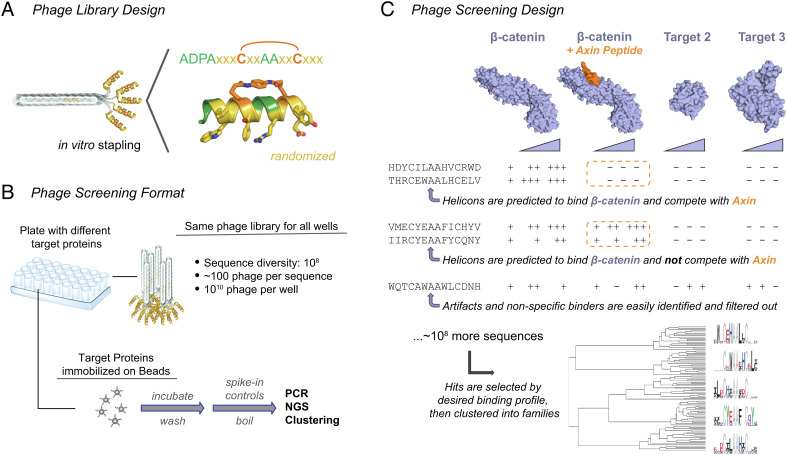
High-throughput and parallelized phage screening identifies Helicons for multiple protein targets in a single experiment. (*A*) Cysteine-stapled Helicons are displayed as N-terminal fusions to the pIII protein on M13 bacteriophage. The two cysteine stapling residues are shown in orange, fixed scaffolding residues are shown in green, and randomized residues are shown in gold. A short glycine-rich linker (not shown) connects the C terminus of the displayed peptide to the phage particle. (*B*) Many phage screens (typically 48 or 96) are run in parallel in multi-well plates. Each well receives approximately 100 copies of each of the 10^8^ library members (10^10^ phage total), allowing direct comparison between all screens. Following binding to target on beads and subsequent washes, phage DNA is directly extracted, amplified, and sequenced. A “spike-in” sequence of known quantity is added to enable the normalization of sequencing reads and quantitative comparison across samples. (*C*) Typical scheme for a screen. Multiple proteins are screened, each under multiple concentrations (*Top*), along with blank beads (not shown). Other controls and comparisons, such as proteins bound to known ligands or proteins with different posttranslational modifications, can be included to enable targeted searches for Helicons with the desired binding mode or mechanism (*Middle*). Following hit selection, sequences are clustered into families, represented here as sequence logos (*Bottom*).

Having identified a suitable phage-compatible cysteine cross-linker, we turned to constructing a naïve α-helical screening library. We designed our library to contain the following features (Fig. 2*A*): An N-terminal cap containing the N-DPAA-C sequence, which can nucleate α-helix formation and which is frequently found at the N terminus of helical segments in protein structures (22, 23); two cysteine residues at i, i+7 positions; three randomized residues flanking each cysteine; a pair of randomized residues toward the inside of the stapled section between the cysteines, two alanine residues at the center of the stapled section (*i, i+3* and *i, i+4*, numbered from the first cysteine) to minimize steric hindrance with the staple; and lastly, a short (8-residue) glycine-rich linker between the last randomized residue and the N terminus of the pIII coat protein.

To randomize the library for display as N-terminal fusions to the M13 bacteriophage pIII protein, we synthesized degenerate primers using an equimolar mix of trimer phosphoroamidites that corresponded to 16 of the 20 naturally occurring amino acids: alanine, arginine, asparagine, aspartic acid, glutamine, glutamic acid, histidine, isoleucine, leucine, methionine, phenyalanine, serine, threonine, tryptophan, tyrosine, and valine. We omitted cysteine and lysine to avoid intramolecular competition with the cross-linking between the two fixed stapling cysteine residues, and omitted proline and glycine to avoid destabilization of the α-helical structure. Phage library members generally matched the expected library design (*SI Appendix*, Fig. S2*B*). With 10 randomized residues total, the theoretical diversity of the library derived from these primers is 16^10^ ≈ 10^12^ possible sequences, and the number of unique sequences after transformation and amplification of the library was estimated to be approximately 10^8^ library members. Hence, the constructed library explores roughly 0.01% of the theoretical sequence space for this design.

Phage display is typically performed by panning a library against a protein target and then eluting and amplifying the bound phage by infection of *Escherichia coli*, followed by additional rounds of selection and amplification (24). This approach has been applied in the context of optimizing helical structures, for example, ‘miniature’ proteins containing α-helical recognition sequences based on the aPP scaffold, or α-helical peptides targeting the Axin-binding site of β-catenin (25, 26). Similarly, in vitro evolution of bicyclic peptides using phage display has identified highly specific binders with affinities in the nanomolar and picomolar range (27). While this typical phage display approach has the advantage of enabling the detection of weak or initially low-abundance binders, it is not practical to perform multiple selections and enrichments on dozens or hundreds of selections in parallel, as we aimed to do here, and it inherently prevents quantitative comparisons between different selections after the first round, because the input phage libraries for each are no longer identical and because of biases in elution efficiency and in growth and infection rates.

Instead, we implemented a single-round approach similar to that reported in ref. 28 to identify bicyclic peptide motifs among phage-display hit sequences. In our approach, the phage are incubated with individual protein targets that have been immobilized on magnetic beads, followed by bead isolation and washing, direct boiling, PCR amplification, and next-generation sequencing (NGS) of the phage DNA followed by hierarchical statistical clustering. These screens are performed in multi-well plates (typically either 48 or 96 screens at a time against a range of protein targets, Fig. 2*B*), and each well receives 10^10^ phage particles from the same 10^8^-member library, such that approximately 100 copies of each library member are initially present in every screening well. Following the binding and washing steps, but prior to boiling and PCR, we add a defined quantity of a known phage sequence spiked into each well. This enables the normalization of NGS counts in each well by dividing the number of reads for each library member by the number of reads for the spike-in, then multiplying the result by the number of spike-in copies that were originally added. This allows the binding of each of the 10^8^ library members to be expressed in terms of eluted phage particles rather than as sequence reads (Fig. 2*C* and *Materials and Methods*).

Since approximately the same number of phage particles were originally added to each well, this treatment permits a quantitative comparison among all samples, for all library members, allowing Helicons to be selected for specific criteria in silico rather than by having to perform actual enrichment or depletion experiments. For example, nonspecific or bead binders can be flagged by comparing hits across multiple proteins as well as against blank beads, since such binders will appear in most or all samples, whereas highly specific binders will appear only in samples corresponding to one target. Similarly, screens can be performed to search for Helicons with specific properties, such as their ability to compete with a known interaction or function of the target; competition for binding sites can be assessed by screening the target in both the presence and absence of a binding partner or ligand, and searching the screening data for Helicons whose phage binding appears affected when the partner or ligand is present (Fig. 2*C*). Since these screens occur in vitro, and dozens or even hundreds can be run in parallel, there is considerable flexibility in screen design and conditions.

Finally, once binders have been selected based on their target-binding and selectivity properties, they are grouped into families of related sequences using hierarchical statistical clustering (Fig. 2*C* and *Materials and Methods*). When the resulting clusters are expressed as a sequence logo, certain positions appear to be highly conserved, with only one or a small number of amino acids being found at that position across all members of the cluster. As revealed by the X-ray cocrystal structures below for the selected targets, these conserved positions are strongly predictive of which residues are forming direct binding interactions with the protein target (see *Discussion*). These residues can therefore be used to define the conserved pharmacophore and predict structure–activity relationships for the cluster (see *Discussion*).

### Discovery of Both Known and De Novo α-Helix Binding Sites on β-Catenin.

To assess the performance of this screening platform, we began with β-catenin, a key hub in the Wnt signaling pathway that is an attractive intracellular target for therapeutic intervention in the many Wnt-driven cancers, such as those with *APC* or *CTNNB1* mutations (29, 30). β-catenin is engaged in numerous functional protein–protein interactions (*SI Appendix*, Fig. S3*A*) that have been difficult to target with traditional small molecules and biologics, but several examples of stapled peptide protein–protein interaction inhibitors based on native binders have been described for β-catenin and other undruggable targets (10, 31–39). Using our platform, we screened β-catenin at multiple concentrations, and in the presence and absence of an Axin-derived Helicon (which binds β-catenin as a short α-helix) and the β-catenin-binding domain of the TCF4 transcription factor. Analysis of five β-catenin-selective binding clusters from this screen revealed two types of binding behavior: binders that competed with both Axin and TCF4, and binders that competed with TCF4 but not with Axin.

**Fig. 3.**
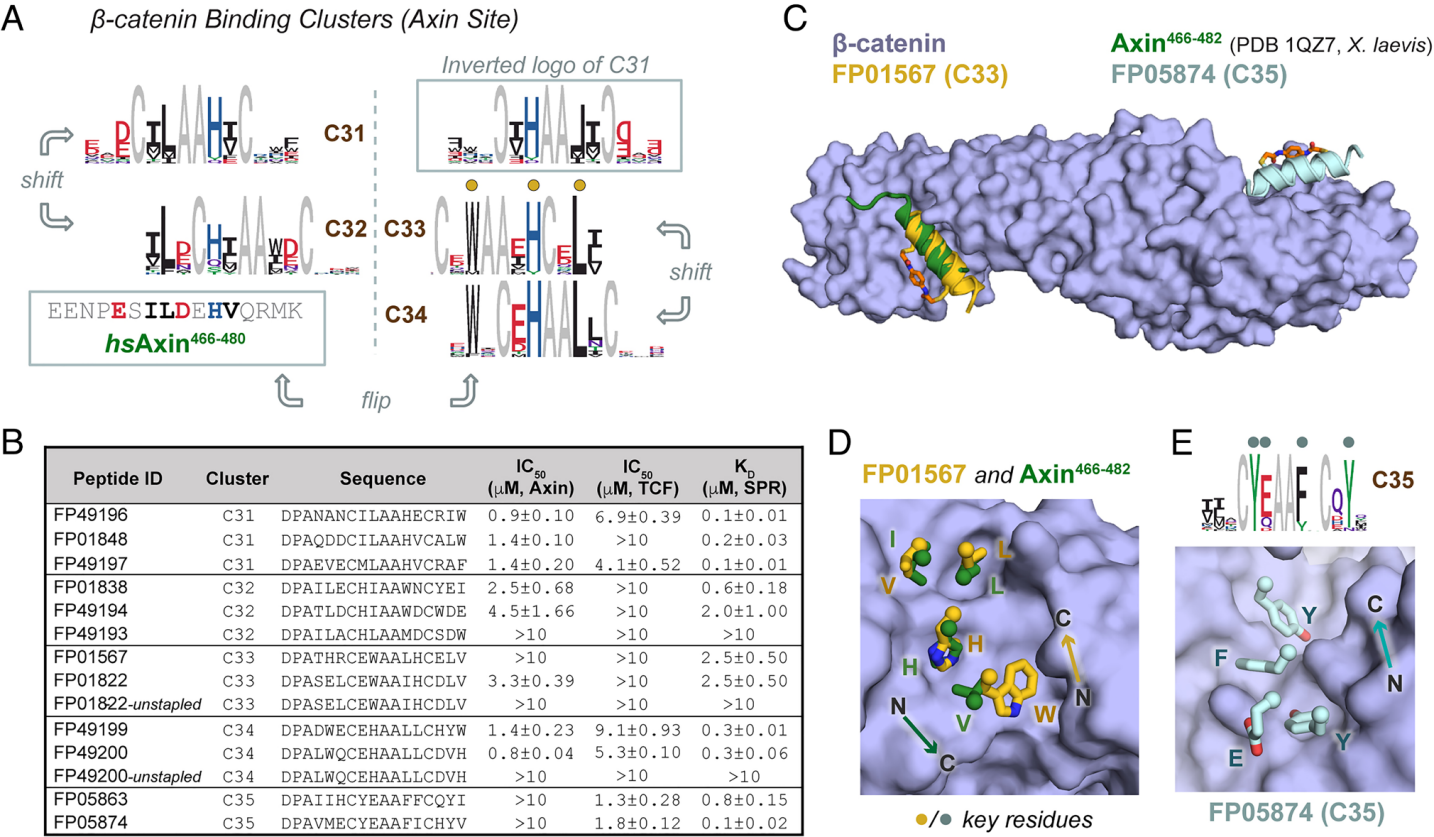
A screen for β-catenin-binding Helicons identifies known binding motifs, variants of known motifs, and binders to novel α-helix binding sites. (*A*) Four β-catenin-binding clusters that were observed to compete with an Axin peptide in the phage screen. Clusters C31 and C32 appear to be “shifts”, as are C33 and C34, and these two pairs are, in turn, “flips” of each other. (*B*) Representative Helicons from C31-34 bind to β-catenin in vitro and compete with β-catenin-Axin binding, consistent with phage screen results. SPR binding data for Helicons FP01567 and FP05874 are shown in *SI Appendix*, Fig. S3*B*. (n = 2 or 3; data are presented as mean ± SD). (*C*) Overlay of X-ray cocrystal structures of FP01567 (C33), FP05874 (C35), and the β-catenin-binding domain of Axin (40). FP01567 binds at the same site as Axin (40), while FP05874 binds closer to the C terminus of the protein. Composite omit maps of these peptides are shown in *SI Appendix*, Fig. S7. (*D*) FP01567 engages the β-catenin surface with similar side-chain contacts as Axin, but with an opposite N-to-C orientation of the α-helix. Key logo residues are marked with dots and indicated as letters. (*E*) Conserved residues in the C35 cluster logo, marked with dots and indicated with letters, correspond to key binding contacts between FP05874 and β-catenin. The N-to-C orientation of the helix is indicated.

When examining clusters that competed with both Axin and TCF4, we were struck by several observations. First, there were pairs of clusters that bore similar patterns of conserved residues, but that were shifted to different positions within the sequence, for example, Clusters C31 and C32 or C33 and C34 (Fig. 3*A*). This suggested to us that the members of these distinct phage clusters may in fact be binding β-catenin in a similar way, but that the pharmacophores are presented in a different position, or register, relative to the position of the staple. We refer to these clusters as “shifts” of each other. Unexpectedly, we also observed clusters whose patterns of conserved residues resembled the conserved residues in other clusters, but were in the reverse orientation (N->C vs. C->N), for example C32 and C33. The implication of such “flips” is that a similar binding mode is achieved from a Helicon whose N- and C termini have been inverted relative to the other clusters, and that the side chains are able to form similar interactions, despite a different presentation angle from the main chain of the Helicon. Remarkably, our cocrystal structure analyses suggest that this is indeed the case (Fig. 3*D*). Finally, we noted that the conserved residues for several of these clusters, such as C31 or C32, bore close resemblance to the α-helical sequence in Axin that is known to bind β-catenin. This suggests that these particular clusters, identified from an unbiased α-helical library, represent a rediscovery of a naturally occurring α-helix binding site and binding mode to β-catenin.

To confirm that the Helicons that appeared to compete with Axin for binding to β-catenin were indeed true binders, we synthesized members of each of the C31 to C35 clusters and tested them in surface plasmon resonance (SPR) and competition fluorescence polarization binding assays. These data indicated binding to β-catenin, as well as competition with both an Axin-derived Helicon and the full β-catenin-binding domain of the TCF4 transcription factor, consistent with the binding behavior predicted from analysis of the screens against β-catenin (Fig. 3*B*, *SI Appendix*, Fig. S3 *A* and *B*, and Dataset S1). A cocrystal structure of β-catenin with FP01567, a Helicon from C33 that was predicted to be a flipped version of the natural Axin sequence, confirmed this binding site, and also confirmed that this Helicon was indeed able to achieve a similar binding mode as Axin but with an inverted/flipped N-to-C α-helical orientation and thus sidechains that were projecting from a different direction (Fig. 3 *C* and *D*). We have since observed frequent shift behavior and occasional flip behavior in many screens across a diverse range of target proteins, suggesting that such behavior may be a common feature of protein surface recognition by Helicons. The crystal structure with FP01567, combined with an alanine-scanning analysis (*SI Appendix*, Fig. S3*C*), further confirmed the importance of the conserved logo residues.

We next turned our attention to those Helicons that competed with TCF, but not Axin, in the phage screen. Biochemical assays performed on members of Cluster C35, which did not resemble any known β-catenin-binding motif, confirmed competition in vitro with TCF but not Axin (*SI Appendix*, Table S2). A cocrystal structure of one member of this cluster, FP05874, showed engagement of β-catenin at a similar site as the naturally occurring β-catenin binder ICAT, but via an isolated α-helical mode that has not, to our knowledge, been observed previously (Fig. 3*D*).

The introduction of all-hydrocarbon staples has been shown to enable a wide range of helical peptides to enter cells (10, 41, 42). To make a preliminary assessment of the potential for the binding sequences we discovered with this platform to be rendered cell-permeable, we selected six Helicons from β-catenin-binding cluster C35 and synthesized a total of 18 Helicons with the cysteine staples replaced by all-hydrocarbon *i, i+7* staples (9). Several of these Helicons maintained measurable binding to β-catenin while also demonstrating cell entry, as assessed by MS quantification of intact peptide in cells after treatment and washing (*SI Appendix*, Table S2).

In summary, this screen for Helicon binders of β-catenin demonstrated the ability of this screening platform to rediscover a natural α-helix binding site and mode, and also to reveal a previously uncharacterized binding site and mode. Comparing the competition data from the screen with biochemical and structural characterization established the platform’s ability to predict binding sites. These data also revealed that families of related clusters could be defined by the similarity of their sequence logos, and that similar binding modes could be achieved by Helicons even when their N-to-C orientations had been reversed. We anticipate that this ability to gain significant functional insight from analysis of the sequencing data will prove useful, given the time and cost savings of eliminating the synthesis and validation of phage hits.

### Multiple α-Helix Binding Sites and Binding Modes on the E3 Ubiquitin Ligase RNF31.

Having established the ability to discover α-helical binders to a protein previously known to bind them, we turned to screening proteins with few or no known α-helical binding sites. We began with the E3 ubiquitin ligase RNF31, which is an integral component of the linear ubiquitin chain assembly complex. RNF31 is a 1,072-amino acid protein comprised of multiple folded and disordered regions. We screened two of the nonenzymatic folded domains, the PUB and UBA domains, each at multiple concentrations and in the presence or absence of a known binding partner (43, 44).

**Fig. 4.**
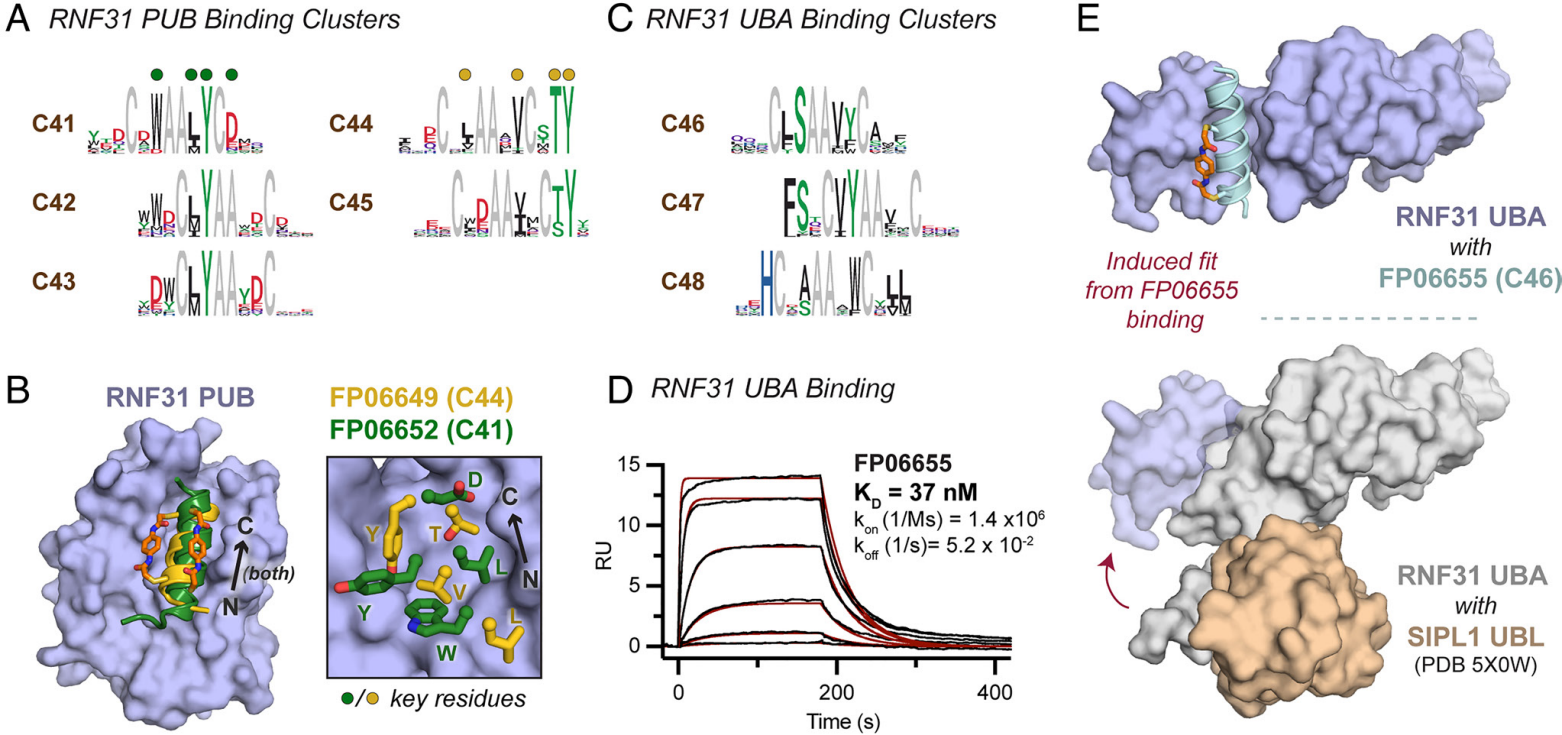
A screen for RNF31-binding Helicons identifies multiple families for both the PUB and UBA domains. (*A*) Five RNF31 PUB binding clusters, which can be grouped into two families. Shifts are observed within both families. (*B*) Overlay of X-ray cocrystal structures of a member from each family of clusters, FP06649 (C44) and FP06652 (C41) (*Left*). Key logo residues are marked with dots and indicated as letters (*Right*). These structures, as composite omit maps, are shown individually in *SI Appendix*, Fig. S7. Despite binding the same α-helix recognition site in the same N-to-C orientation, the two Helicons engage the protein surface with completely different sidechains. (*C*) Three RNF31 UBA binding clusters, which can be grouped into two families. C46 and C47 appear to be shifts of each other. (*D*) Surface plasmon resonance (SPR, Biacore) binding of FP06655 (C46) to the RNF31 UBA domain. (*E*) X-ray cocrystal structure of FP06655 bound to the RNF31 UBA domain, with a composite omit map shown in *SI Appendix*, Fig. S7. A significant conformational rearrangement is observed (lilac overlay) relative to the previously reported cocrystal structure of the RNF31 UBA domain (gray) with its binding partner Sharpin/SIPL1 (peach) (45).

Screens for both domains afforded multiple distinct clusters of binders, and we again observed several instances of shift clusters. Most hits for the RNF31 PUB domain could be grouped into two families of related clusters (C41 to C43 and C44 to C45), both of which competed with the natural binding partner Otulin, and both of which appeared to contain a critical tyrosine residue – also found in Otulin – but that had otherwise dissimilar logos (Fig. 4*A* and *SI Appendix*, Fig. S4 *A* and *B*). Upon solving X-ray cocrystal structures of member from a representative cluster of each family, we found that two Helicons, FP06649 from Cluster C44 and FP06652 from C41, bound the same site at the protein surface, but that they engaged this site using completely different binding modes, with the critical tyrosine residues oriented differently (Fig. 4*B*). This observation was our first structural evidence of the ability of Helicons to identify distinct binding solutions to a common site, which we have since observed for several other target proteins.

Binders to the RNF31 UBA domain, all of which compete with its partner Sharpin/SIPL1 in the phage screen, could also be grouped into at least two families. One of these families, comprised of Cluster C46 and C47, was characterized by a shared compact pharmacophore containing conserved leucine/phenylalanine, serine, valine, and tyrosine residues that was observed in two shifted registers (Fig. 4*C*). Biochemical characterization of members from C46 revealed that several Helicons bound with mid-nanomolar affinity (Fig. 4*D*). However, when we analyzed a cocrystal structure of one of these Helicons, FP06655, in complex with the RNF31 UBA domain, we observed an unexpectedly large conformational rearrangement when compared with the cocrystal structure of the RNF31 UBA domain in complex with its natural binding partner Sharpin/SIPL1 (Fig. 4*E*). This rearrangement involves a significant rotation of the bundle of three N-terminal α-helices of the RNF31 UBA domain to form a groove in which the conserved leucine/phenylalanine and valine residues of FP06655 engage one wall of the groove, and the conserved serine and tyrosine residues engage the opposite wall, thereby stabilizing the RNF31 UBA domain in this induced fit. To confirm that this binding mode was not an artifact of crystallization, we showed that Sharpin UBL indeed competes with 5FAM-labeled FP06655 in solution (*SI Appendix*, Fig. S4*C*). Together, these data illustrate the ability of Helicon binding to cause extreme induced fits, another feature of Helicon–protein recognition that we have now observed in several contexts.

### Orthosteric and Allosteric Binding to Enzymes.

To assess the ability of Helicons to bind and modulate the function of enzymes, we screened the cyclin-dependent kinase CDK2, involved in the regulation of the cell cycle, and also the peptidyl-prolyl cis-trans isomerase Cyclophilin A (PPIA), which binds non-α-helical peptide substrates and catalyzes the cis-trans isomerization of proline residues.

CDK2 is a protein target of high therapeutic interest due to its role in cell cycle progression and its implication in CyclinE1-mediated resistance to CDK4/6-inhibitor treated cancers (46, 47), but it has historically been challenging to develop CDK2-selective inhibitors, particularly using small molecules, due to the close similarity of CDK2 with other CDK family members, particularly CDK1. Whereas the ATP-binding pockets of CDK2 and CDK1 are highly similar, their surfaces share significantly less sequence identity, allowing the possibility that Helicons that engage the CDK2 surface may be capable of differentiating between the two family members.

**Fig. 5.**
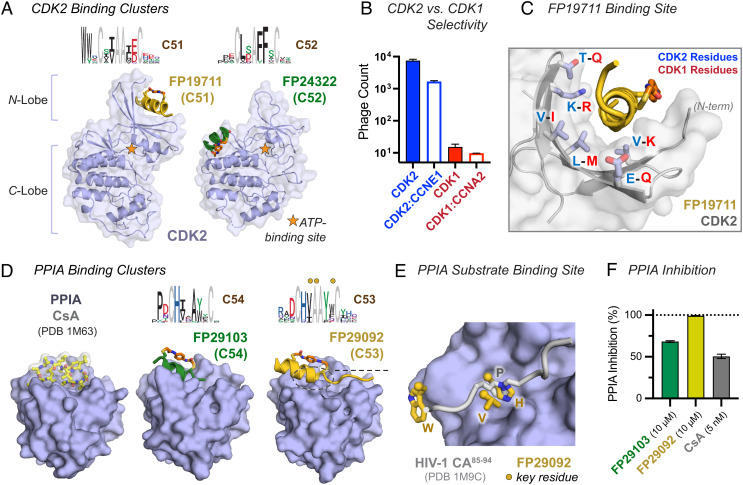
Screens of CDK2 and PPIA identify Helicons with high CDK2 selectivity and inhibitors of PPIA-CsA binding and PPIA catalytic activity. (*A*) Phage cluster logos for two CDK2 binding clusters, and X-ray cocrystal structures of one member from each, FP19711 (C51) and FP24322 (C52). These two Helicons bind distinct sites on the CDK2 surface. Composite omit maps are shown in *SI Appendix*, Fig. S7. (*B*) FP19711 shows selectivity for CDK2 and the CDK2–CCNE1 complex over CDK1 or the CDK1–CCNA2 complex on phage. (n = 2; data are presented as mean ± SD) (*C*) The FP19711 binding site on CDK2 contains several amino acid differences relative to CDK1. (*D*) Two related clusters that bind PPIA (C54 and C53 with their logos shown), and X-ray cocrystal structures of one member from each: FP29103 (C54), discovered from a truncated version of our standard phage library) and FP29092 (C53). Closer views of the structures are shown in *SI Appendix*, Fig. S7 for these two Helicons and FP29102 from C54. These Helicons recognize a similar site as natural product CsA (48) (*Left*). (*E*) Helicon binding overlaps with the PPIA substrate-binding site [example from the HIV capsid protein shown (49)]. Key logo residues are marked with dots and indicated as letters. (*F*) PPIA-binding Helicons inhibit peptidyl-prolyl cis-trans isomerase activity, similar to the control CsA but with much higher IC_50_ (n = 3; data are presented as mean ± SD).

Upon performing a phage screen, we found two distinct clusters of Helicons that bound both CDK2 and CDK2 in complex with its partner CyclinE1, but not to CDK1 or CDK1 in complex with its partner CyclinA2 (C51 and C52). We solved cocrystal structures of Helicons from each of these clusters, FP19711 from Cluster C51 and FP24322 from C52, revealing two allosteric binding sites with respect to the ATP pocket (Fig. 5*A* and *SI Appendix*, Fig. S5*A*). A FP19711-CDK2 costructure indicated that the N-terminal and C-terminal-most peptide residues are not directly involved in the CDK2 interaction. Truncation of both the N- and C termini to generate FP33215 improved the affinity of FP19711 to 300 nM (*SI Appendix*, Fig. S5*B*). We also confirmed biochemically that FP19711 does not compete with ATP, and could still bind to CDK2 in the presence of other ATP-competitive CDK2-binding proteins (*SI Appendix*, Fig. S5 *C* and *D*). Consistent with the selectivity observed in the phage screen (Fig. 5*B*), the FP19711 binding site is found at a region on the CDK2 surface where there is high divergence from CDK1 (Fig. 5*C*). These results highlight the utility of exploiting surface binding to achieve selectivity between closely related proteins when selectivity is difficult to achieve with small-molecule ligands.

Peptidyl-prolyl cis-trans isomerases (PPIases) are a family of enzymes that recognize a diverse range of proline-containing polypeptide substrates, and are targets of the “trimerizer” natural products CsA and Sanglifehrin A that bind PPIA to form ternary complexes with the proteins Calcineurin and IMPDH2, respectively (50, 51). A Helicon screen for PPIA binders afforded a pair of shifted clusters that competed with CsA both in the phage screen and in in vitro biochemical assays. Cocrystal structures of Helicons from both Clusters C53 and C54 confirmed that they bound the site ordinarily occupied by peptide substrates or natural products, providing a second instance where the screening platform was able to identify an α-helical binding solution to a site ordinarily recognized by peptides in a non-α-helical conformation (Fig. 5 *D* and *E* and *SI Appendix*, Fig. S5 *E*–*G*). A PPIase assay showed that these Helicons inhibit PPIA activity, as expected given their orthosteric binding mode, demonstrating the ability of Helicons to directly block enzymatic function (Fig. 5*F*).

### Inhibition and Induced Dimerization of Programmed Cell Death 1 Ligand 1 (PD-L1).

**Fig. 6.**
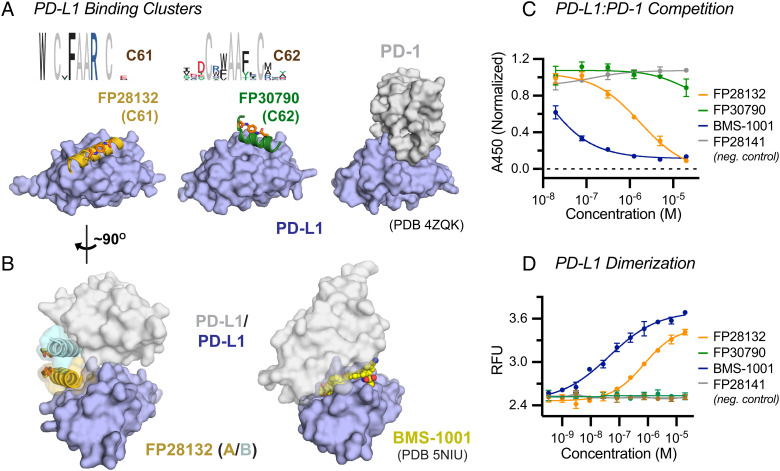
A screen for PD-L1-binding Helicons identifies inhibitors of the PD-L1:PD-1 interaction and inducers of PD-L1 dimerization. (*A*) Phage cluster logos for two PD-L1 binding clusters, and X-ray cocrystal structures of one member from each, FP28132 (C61) and FP30790 (C62). Both Helicons recognize sites that overlap with the PD-1 binding site on PD-L1. Composite omit maps of the helical structures are shown in *SI Appendix*, Fig. S7 for these two Helicons and for FP28135 and FP28136. (*B*) The FP28132-PD-L1 complex appears to dimerize in the crystal lattice, in a manner distinct from the previously reported PD-L1 dimerizer BMS-1001. (*C*) FP28132 and BMS-1001, but not the closely matched FP28132-derived control FP28141, inhibits PD-L1:PD-1 binding in a competition ELISA assay (n = 2; data are presented as mean ± SD) and (*D*) induce PD-L1 dimerization in solution as assayed by TR-FRET (n = 3 for FP peptides and n = 2 for BMS-1001; data are presented as mean ± SD).

Having established that Helicons could be discovered for a variety of intracellular proteins, we lastly asked whether the same was true for extracellular domains (ECDs). We selected the ECD of the transmembrane protein PD-L1, which binds to the ECD of Programmed cell death protein 1 (PD-1) to suppress T cell function (52), and which has not, to our knowledge, been shown to bind α-helices. A screen for PD-L1 binders afforded two clusters of Helicons (C61 and C62) that were both predicted to compete with PD-1 on the basis of their on-phage competition behavior (Fig. 6*A*). In vitro competition enzyme-linked immunoassay (ELISA) confirmed this prediction for FP28132 from Cluster C61 (Fig. 6*C*). FP30790 from Cluster C62 is a relatively weak binder and did not show competition in the ELISA format, but competition SPR confirmed that PD1 blocks its binding to PD-L1 (*SI Appendix*, Fig. S6*A*). Cocrystal structures of PD-L1 with these two Helicons showed that the two clusters both engage the PD-1 binding surface of PD-L1, but occupy distinct α-helical sites on it (Fig. 6*B*).

The asymmetric unit of the FP28132 / PD-L1 cocrystal structure contains a symmetric dimer of two FP28132 / PD-L1 complexes (Fig. 6*B*). We have also structurally characterized two other Helicons, FP28135 and FP28136 derived from the same PD-L1-binding phage cluster, and observed a similar assembly (*SI Appendix*, Fig. S6 *B* and *C*). This revealed an extensive series of contacts between both the two Helicon protomers and the two PD-L1 protomers (*SI Appendix*, Fig. S6*D*). To assess whether this is an indication that the FP28132 / PD-L1 complex is a dimer in solution, we performed analytical size-exclusion chromatography (*SI Appendix*, Fig. S6*E*) and time-resolved fluorescence energy transfer (TR-FRET) experiments with PD-L1 in the presence and absence of FP28132, FP30790, a non-PD-L1 binding mutant of FP28132 that differs by one residue (FP28141), and BMS-1001, a small-molecule PD-L1 binder that has also been shown to induce protein dimerization. We observed clear evidence of PD-L1 dimerization in the presence of both BMS-1001 and FP28132, but not in the presence of FP30790 or FP28141 (Fig. 6*D*). Taken together, these findings demonstrate the ability to discover multiple α-helix binding sites to a relatively small ECD that was not previously known to have any, as well as the ability of Helicons to induce protein dimerization.

## Discussion

The field of synthetically stabilized α-helical polypeptides as a new drug modality has generated considerable interest and research activity since its inception more than two decades ago (9), with two such molecules having entered the clinic, one of which is currently in phase II clinical trials (14). However, the lack of a general method for discovering α-helical binders to proteins de novo has limited research in this field to primarily those proteins and binding sites for which α-helices had already been discovered, which in most cases has been limited to their naturally occurring α-helical binding partners. This has constrained the protein targets and inhibitory mechanisms that could be pursued, and has also restricted the number of distinct chemical series available to medicinal chemists for developing drug candidates.

Here, we have described a platform that allows for the swift, unbiased screening of large Helicon libraries using phage display. We show that this platform is able to identify previously unknown α-helix binding sites on a range of protein folds and types, including a transcriptional regulator (β-catenin), two structurally dissimilar domains from an E3 ubiquitin ligase (RNF31), a kinase (CDK2), a peptidyl-prolyl cis-trans isomerase (PPIA), and an extracellular receptor (PD-L1). X-ray cocrystal structures indicate that many of these sites are located on surfaces not previously known to bind α-helices. Biochemical and structural experiments demonstrate diverse functional impacts by the Helicons which bind these sites, including inhibition of protein–protein interactions, inhibition of enzymatic activity, induced conformational rearrangement, and induced dimerization. We have since successfully performed screens with a wide range of small molecule, peptidic, and protein binding partners, and have also compared phosphorylated vs. nonphosphorylated, glycosylated vs. nonglycosylated, apo vs. ligand-bound, mouse vs. human, and monomeric vs. multimeric proteins. We also envision several possible modifications to the screening approach reported here that might facilitate the identification of stronger binders than the low-micromolar and high-nanomolar affinities we found for most targets. One or more panning rounds prior to the multi-well screen could be introduced to enrich low-abundance library members and provide clearer signals to select binders with, and the use of monovalent phagemid systems would avoid the avidity effects of the multivalent phage system that can complicate the interpretation of apparent on-phage affinities.

A critical feature of Helicons is their intrinsic cloaking of the main-chain amide protons that are an impediment to membrane penetration for non-α-helical peptides. We inspected the 14 cocrystal structures reported in this paper to confirm that the Helicons discovered with our phage screening platform were indeed α-helical in structure, and that they did not have exposed main-chain amide protons and were not using main-chain amide bonds for protein target recognition. In all cases, the Helicons were α-helical in the region of the molecule involved in engaging the target, with few examples of fraying at the ends of Helicons outside of the stapled region (*SI Appendix*, Fig. S7). We also did not observe significant exposure of amide protons in any Helicon structure, except those at the N terminus of each Helicon, which is expected due to the absence of a corresponding carbonyl to pair with (*SI Appendix*, Fig. S7). Inspection of binding contacts between Helicons and their targets also revealed no examples of protein surface engagement by any Helicon main-chain amides. While cocrystal structures are only capable of providing a snapshot of Helicon–protein recognition, and cannot capture all of the interactions that will occur in solution, these findings suggest that the Helicons identified with this platform do not heavily rely on main-chain interactions to bind their targets.

An important step in the early stages of medicinal chemistry optimization campaigns is the establishment of structure–activity relationships to identify which features of a compound are critical for binding and activity (53). In the absence of a cocrystal structure or other direct structural information, establishing structure–activity relationships typically requires extensive empirical testing of compound analogs. Due to the relatively large number of Helicons in many of the clusters discovered in this phage platform, we wondered whether there was sufficient information in the phage cluster logos to predict which amino acids were involved in target binding. As detailed above, comparison of cluster logos with cocrystal structures revealed that in almost all cases, highly conserved amino acids were directly participating in protein binding (Figs. 3*E*, 4*B*, and 5 *C*–*E*). An alanine-mutagenesis scan of the β-catenin-binding Helicon FP01567 showed good agreement between binding data of alanine mutants and residues predicted to be important for binding based on both structural and logo-based analysis (*SI Appendix*, Fig. S3). Although a complete evaluation of this hypothesis would require further biochemical and biochemical studies of many additional series, these findings provide a preliminary indication that cluster logo information may prove useful in the early stages of Helicon optimization, particularly when structural data are lacking.

In sum, we have developed a general and rapid screening method for discovering α-helical binders to proteins, and demonstrated its scope by reporting screening data for six structurally diverse protein domains, with biochemical validation of Helicons from 20 unique clusters and cocrystal structures of Helicons from 14 of those. We expect that this platform will prove useful both for the development of new chemical tools for interrogating protein function and for the development of therapeutics for target proteins that remain out of reach of traditional drug modalities.

## Materials and Methods

Cross-linked (stapled) peptides were generated using standard Fmoc-based solid-phase peptide synthesis followed by in-solution cysteine bisalkylation. CD spectroscopy was performed to compare stapled and unstapled peptide pairs. Protocols for phage library construction largely followed guidelines in the New England Biolabs Ph.D. Peptide Display Cloning system kit. The resulting libraries were stapled following phage purification, and both Sanger and NGS were used to assess library quality. Single-round phage library screening was performed using biotinylated proteins bound to streptavidin magnetic beads followed by washing and NGS of target-bound library members. Standard biochemical methods were used for production and purification of target proteins recombinantly expressed in bacterial, insect, or mammalian cells. SPR and Fluorescence Polarization competition were used to assess the ability of Helicons to bind to target proteins and to compete with binding partners. The fraction of cell-associated Helicons was quantitated using MS analysis of HEK293 cells following incubation with Helicons and washing. Nucleotide competition of CDK2-binding Helicons was assessed using a fluorescence anisotropy-based assay, inhibition of PPIA was assessed using a colorimetric assay, inhibition of PD-1/PD-L1 interactions was assessed by ELISA, and PD-L1 dimerization was assessed by TR-FRET. X-ray crystallography and structure determination were done using standard methods. Details of all materials and methods are available in *SI Appendix*, *SI Materials and Methods*.

## Supplementary Material

Appendix 01 (PDF)

Dataset S01 (XLSX)

Dataset S02 (XLSX)

## Data Availability

All study data and associated protocols are included in the article. X-ray crystallography data have been deposited in Protein Data Bank [PDB IDs: 7UWI (54), 7UWO (55), 7UX5 (56), 7UXI (57), 7UXJ (58), 7UXK (59), 7UXM (60), 7UXN (61), 7UXO (62), 7UXP (63), 7UXQ (64), 7UY2 (65), 7UYJ (66), and 7UYK (67)].

## References

[1] Z. Qian, P. G. Dougherty, D. Pei, Targeting intracellular protein–protein interactions with cell-permeable cyclic peptides. Curr. Opin. Chem. Biol. **38**, 80–86 (2017). 28388463 10.1016/j.cbpa.2017.03.011 PMC5474178

[2] P. G. Dougherty, A. Sahni, D. Pei, Understanding cell penetration of cyclic peptides. Chem. Rev. **119**, 10241–10287 (2019). 31083977 10.1021/acs.chemrev.9b00008 PMC6739158

[3] T. K. Sawyer , Macrocyclic α helical peptide therapeutic modality: A perspective of learnings and challenges. Bioorg. Med. Chem. **26**, 2807–2815 (2018). 29598901 10.1016/j.bmc.2018.03.008

[4] C. L. Ahlbach , Beyond cyclosporine A: Conformation-dependent passive membrane permeabilities of cyclic peptide natural products. Future Med. Chem. **7**, 2121–2130 (2015). 26067057 10.4155/fmc.15.78 PMC5186412

[5] A. T. Bockus , Probing the physicochemical boundaries of cell permeability and oral bioavailability in lipophilic macrocycles inspired by natural products. J. Med. Chem. **58**, 4581–4589 (2015). 25950816 10.1021/acs.jmedchem.5b00128

[6] J. L. Hickey , Passive membrane permeability of macrocycles can be controlled by exocyclic amide bonds. J. Med. Chem. **59**, 5368–5376 (2016). 27120576 10.1021/acs.jmedchem.6b00222

[7] A. F. B. Räder, F. Reichart, M. Weinmüller, H. Kessler, Improving oral bioavailability of cyclic peptides by N-methylation. Bioorg. Med. Chem. **26**, 2766–2773 (2018). 28886995 10.1016/j.bmc.2017.08.031

[8] S. H. White, W. C. Wimley, Membrane protein folding and stability: Physical principles. Annu. Rev. Biophys. Biomol. Struct. **28**, 319–365 (1999). 10410805 10.1146/annurev.biophys.28.1.319

[9] C. E. Schafmeister, J. Po, G. L. Verdine, An all-hydrocarbon cross-linking system for enhancing the helicity and metabolic stability of peptides. J. Am. Chem. Soc. **122**, 5891–5892 (2000).

[10] L. D. Walensky , Activation of apoptosis in vivo by a hydrocarbon-stapled BH3 helix. Science **305**, 1466–1470 (2004). 15353804 10.1126/science.1099191 PMC1360987

[11] G. L. Verdine, L. D. Walensky, The challenge of drugging undruggable targets in cancer: Lessons learned from targeting BCL-2 family members. Clin. Cancer Res. **13**, 7264–7270 (2007). 18094406 10.1158/1078-0432.CCR-07-2184

[12] D. P. Fairlie, A. D. de Araujo, Stapling peptides using cysteine crosslinking. Peptide Sci. **106**, 843–852 (2016). 10.1002/bip.22877 27178225

[13] Y. S. Chang , Stapled α−helical peptide drug development: A potent dual inhibitor of MDM2 and MDMX for p53-dependent cancer therapy. Proc. Natl. Acad. Sci. U.S.A. **110**, E3445–E3454 (2013). 23946421 10.1073/pnas.1303002110 PMC3767549

[14] M. N. Saleh , Phase 1 trial of ALRN-6924, a dual inhibitor of MDMX and MDM2, in patients with solid tumors and lymphomas bearing wild-type TP53. Clin. Cancer Res. **27**, 5236–5247 (2021). 34301750 10.1158/1078-0432.CCR-21-0715 PMC9401461

[15] D. Fujiwara , Chemical modification of phage-displayed helix-loop-helix peptides to construct kinase-focused libraries. Chembiochem **22**, 3406–3409 (2021). 34605137 10.1002/cbic.202100450 PMC9297947

[16] M. Hashimoto, T. Miki, I. V. Chang, H. Tsutsumi, H. Mihara, Selection of fluorescent biosensors against galectin-3 from an NBD-modified phage library displaying designed α-helical peptides. Bioorg. Med. Chem. Lett. **37**, 127835 (2021). 33556574 10.1016/j.bmcl.2021.127835

[17] T. R. Oppewal, I. D. Jansen, J. Hekelaar, C. Mayer, A strategy to select macrocyclic peptides featuring asymmetric molecular scaffolds as cyclization units by phage display. J. Am. Chem. Soc. **144**, 3644–3652 (2022). 35171585 10.1021/jacs.1c12822 PMC8895403

[18] C. Heinis, T. Rutherford, S. Freund, G. Winter, Phage-encoded combinatorial chemical libraries based on bicyclic peptides. Nat. Chem. Biol. **5**, 502–507 (2009). 19483697 10.1038/nchembio.184

[19] M. R. Jafari , Discovery of light-responsive ligands through screening of a light-responsive genetically encoded library. ACS Chem. Biol. **9**, 443–450 (2014). 24195775 10.1021/cb4006722

[20] T. Anananuchatkul, H. Tsutsumi, T. Miki, H. Mihara, hDM2 protein-binding peptides screened from stapled α-helical peptide phage display libraries with different types of staple linkers. Bioorg. Med. Chem. Lett. **30**, 127605 (2020). 33038548 10.1016/j.bmcl.2020.127605

[21] H. Dotter, M. Boll, M. Eder, A.-C. Eder, Library and post-translational modifications of peptide-based display systems. Biotechnol. Adv. **47**, 107699 (2021). 33513435 10.1016/j.biotechadv.2021.107699

[22] M. K. Kim, Y. K. Kang, Positional preference of proline in α-helices. Protein Sci. **8**, 1492–1499 (1999). 10422838 10.1110/ps.8.7.1492 PMC2144370

[23] R. Aurora, G. D. Rosee, Helix capping. Protein Sci. **7**, 21–38 (1998). 9514257 10.1002/pro.5560070103 PMC2143812

[24] G. P. Smith, V. A. Petrenko, Phage display. Chem. Rev. **97**, 391–410 (1997). 11848876 10.1021/cr960065d

[25] P. Diderich , Phage selection of chemically stabilized α-helical peptide ligands. ACS Chem. Biol. **11**, 1422–1427 (2016). 26929989 10.1021/acschembio.5b00963

[26] J. W. Chin, R. M. Grotzfeld, M. A. Fabian, A. Schepartz, Methodology for optimizing functional miniature proteins based on avian pancreatic polypeptide using phage display. Bioorg. Med. Chem. Lett. **11**, 1501–1505 (2001). 11412969 10.1016/s0960-894x(01)00139-1

[27] S. Chen, C. Heinis, Peptide libraries, methods and protocols. Methods Mol. Biol. **1248**, 119–137 (2014). 10.1007/978-1-4939-2020-4_9 25616330

[28] I. R. Rebollo, M. Sabisz, V. Baeriswyl, C. Heinis, Identification of target-binding peptide motifs by high-throughput sequencing of phage-selected peptides. Nucleic Acids Res. **42**, e169 (2014). 25348396 10.1093/nar/gku940 PMC4267670

[29] M. J. Patrice , Activation of beta -catenin-Tcf signaling in colon cancer by mutations in beta -catenin or APC. Science **275**, 1787–1790 (1997). 9065402 10.1126/science.275.5307.1787

[30] Z. Ya, W. Xin, Targeting the Wnt/β-catenin signaling pathway in cancer. J. Hematol. Oncol. **13**, 165 (2020). 33276800 10.1186/s13045-020-00990-3 PMC7716495

[31] F. Bernal , A stapled p53 helix overcomes HDMX-mediated suppression of p53. Cancer Cell **18**, 411–422 (2010). 21075307 10.1016/j.ccr.2010.10.024 PMC3050021

[32] C. Phillips , Design and structure of stapled peptides binding to estrogen receptors. J. Am. Chem. Soc. **133**, 9696–9699 (2011). 21612236 10.1021/ja202946k

[33] T. N. Grossmann , Inhibition of oncogenic Wnt signaling through direct targeting of β-catenin. Proc. Natl. Acad. Sci. U.S.A. **109**, 17942–17947 (2012). 23071338 10.1073/pnas.1208396109 PMC3497784

[34] K. Takada , Targeted disruption of the BCL9/β-catenin complex inhibits oncogenic Wnt signaling. Sci. Transl. Med. **4**, 148ra117 (2012). 10.1126/scitranslmed.3003808 PMC3631420 22914623

[35] L. Nevola , Light-regulated stapled peptides to inhibit protein-protein interactions involved in clathrin-mediated endocytosis. Angew. Chem. Int. Ed. Engl. **52**, 7704–7708 (2013). 23775788 10.1002/anie.201303324

[36] J. Spiegel , Direct targeting of Rab-GTPase–effector interactions. Angew. Chem. Int. Ed. Engl. **53**, 2498–2503 (2014). 24481744 10.1002/anie.201308568

[37] I. de Paola , Cullin3 - BTB interface: A novel target for stapled peptides. PLoS One **10**, e0121149 (2015). 25848797 10.1371/journal.pone.0121149 PMC4388676

[38] T. Misawa, Y. Demizu, M. Kawamura, N. Yamagata, M. Kurihara, Structural development of stapled short helical peptides as vitamin D receptor (VDR)–coactivator interaction inhibitors. Bioorg. Med. Chem. **23**, 1055–1061 (2015). 25637122 10.1016/j.bmc.2015.01.007

[39] L. Dietrich , Cell permeable stapled peptide inhibitor of Wnt signaling that targets β-catenin protein-protein interactions. Cell Chem. Biol. **24**, 958–968.e5 (2017). 28757184 10.1016/j.chembiol.2017.06.013

[40] Y. Xing, W. K. Clements, D. Kimelman, W. Xu, Crystal structure of a β-catenin/Axin complex suggests a mechanism for the β-catenin destruction complex. Genes Dev. **17**, 2753–2764 (2003). 14600025 10.1101/gad.1142603 PMC280624

[41] A. Steinauer , HOPS-dependent endosomal fusion required for efficient cytosolic delivery of therapeutic peptides and small proteins. Proc. Natl. Acad. Sci. U.S.A. **116**, 512–521 (2019). 30610181 10.1073/pnas.1812044116 PMC6329960

[42] A. Peier , NanoClick: A high throughput, target-agnostic peptide cell permeability assay. ACS Chem. Biol. **16**, 293–309 (2021). 33539064 10.1021/acschembio.0c00804

[43] V. Schaeffer , Binding of OTULIN to the PUB domain of HOIP controls NF-κB signaling. Mol. Cell **54**, 349–361 (2014). 24726327 10.1016/j.molcel.2014.03.016

[44] F. Tokunaga , SHARPIN is a component of the NF-κB-activating linear ubiquitin chain assembly complex. Nature **471**, 633–636 (2011). 21455180 10.1038/nature09815

[45] J. Liu , Structural insights into SHARPIN-mediated activation of HOIP for the linear ubiquitin chain assembly. Cell Rep. **21**, 27–36 (2017). 28978479 10.1016/j.celrep.2017.09.031

[46] T. Chohan, H. Qian, Y. Pan, J.-Z. Chen, Cyclin-dependent kinase-2 as a target for cancer therapy: Progress in the development of CDK2 inhibitors as anti-cancer agents. Curr. Med. Chem. **22**, 237–263 (2014). 10.2174/0929867321666141106113633 25386824

[47] E. S. Knudsen, A. K. Witkiewicz, The strange case of CDK4/6 inhibitors: Mechanisms, resistance, and combination strategies. Trends Cancer **3**, 39–55 (2017). 28303264 10.1016/j.trecan.2016.11.006 PMC5347397

[48] Q. Huai , Crystal structure of calcineurin–cyclophilin–cyclosporin shows common but distinct recognition of immunophilin–drug complexes. Proc. Natl. Acad. Sci. U.S.A. **99**, 12037–12042 (2002). 12218175 10.1073/pnas.192206699 PMC129394

[49] B. R. Howard, F. F. Vajdos, S. Li, W. I. Sundquist, C. P. Hill, Structural insights into the catalytic mechanism of cyclophilin A. Nat. Struct. Mol. Biol. **10**, 475–481 (2003). 10.1038/nsb927 12730686

[50] G. Fischer, T. Aumüller, Regulation of peptide bond cis/trans isomerization by enzyme catalysis and its implication in physiological processes. Rev. Physiol. Biochem. Pharmacol. **148**, 105–150 (2003). 12698322 10.1007/s10254-003-0011-3

[51] J. Liu , Calcineurin is a common target of cyclophilin-cyclosporin A and FKBP-FK506 complexes. Cell **66**, 807–815 (1991). 1715244 10.1016/0092-8674(91)90124-h

[52] M. E. Keir, M. J. Butte, G. J. Freeman, A. H. Sharpe, PD-1 and its ligands in tolerance and immunity. Annu. Rev. Immunol. **26**, 677–704 (2008). 18173375 10.1146/annurev.immunol.26.021607.090331 PMC10637733

[53] P. Imming, “Medicinal chemistry: Definitions and objectives, drug activity phases, drug classification systems” in The Practice of Medicinal Chemistry, C. Wermuth, D. Aldous, P. Raboisson, D. Rognan, Eds. (Academic Press, ed. 4, 2015).

[54] M. Brennan , De novo mapping of α-helix recognition sites on protein surfaces using unbiased libraries. PDB. https://www.rcsb.org/structure/7UWI. Deposited 3 May 2022. 10.1073/pnas.2210435119 PMC9907135 36534810

[55] S. Agarwal , De novo mapping of α-helix recognition sites on protein surfaces using unbiased libraries. PDB. https://www.rcsb.org/structure/7UWO. Deposited 3 May 2022. 10.1073/pnas.2210435119 PMC9907135 36534810

[56] S. Agarwal , De novo mapping of α-helix recognition sites on protein surfaces using unbiased libraries. PDB. https://www.rcsb.org/structure/7UX5. Deposited 5 May 2022 10.1073/pnas.2210435119 PMC9907135 36534810

[57] K. Li , De novo mapping of α-helix recognition sites on protein surfaces using unbiased libraries. PDB. https://www.rcsb.org/structure/7UXI. Deposited 5 May 2022. 10.1073/pnas.2210435119 PMC9907135 36534810

[58] K. Li , De novo mapping of α-helix recognition sites on protein surfaces using unbiased libraries. PDB. https://www.rcsb.org/structure/7UXJ. Deposited 5 May 2022. 10.1073/pnas.2210435119 PMC9907135 36534810

[59] K. Li , De novo mapping of α-helix recognition sites on protein surfaces using unbiased libraries. PDB. https://www.rcsb.org/structure/7UXK. Deposited 5 May 2022. 10.1073/pnas.2210435119 PMC9907135 36534810

[60] K. Li , De novo mapping of α-helix recognition sites on protein surfaces using unbiased libraries. PDB. https://www.rcsb.org/structure/7UXM. Deposited 5 May 2022. 10.1073/pnas.2210435119 PMC9907135 36534810

[61] K. Li , De novo mapping of α-helix recognition sites on protein surfaces using unbiased libraries. PDB. https://www.rcsb.org/structure/7UXN. Deposited 5 May 2022. 10.1073/pnas.2210435119 PMC9907135 36534810

[62] K. Li , De novo mapping of α-helix recognition sites on protein surfaces using unbiased libraries. PDB. https://www.rcsb.org/structure/7UXO. Deposited 5 May 2022. 10.1073/pnas.2210435119 PMC9907135 36534810

[63] K. Li , De novo mapping of α-helix recognition sites on protein surfaces using unbiased libraries. PDB. https://www.rcsb.org/structure/7UXP. Deposited 5 May 2022. 10.1073/pnas.2210435119 PMC9907135 36534810

[64] K. Li , De novo mapping of α-helix recognition sites on protein surfaces using unbiased libraries. PDB. https://www.rcsb.org/structure/7UXQ. Deposited 5 May 2022. 10.1073/pnas.2210435119 PMC9907135 36534810

[65] S. Agarwal , De novo mapping of α-helix recognition sites on protein surfaces using unbiased libraries. PDB. https://www.rcsb.org/structure/7UY2. Deposited 6 May 2022. 10.1073/pnas.2210435119 PMC9907135 36534810

[66] S. Agarwal , De novo mapping of α-helix recognition sites on protein surfaces using unbiased libraries. PDB. https://www.rcsb.org/structure/7UYJ. Deposited 6 May 2022. 10.1073/pnas.2210435119 PMC9907135 36534810

[67] S. Agarwal , De novo mapping of α-helix recognition sites on protein surfaces using unbiased libraries. PDB. https://www.rcsb.org/structure/7UYK. Deposited 6 May 2022. 10.1073/pnas.2210435119 PMC9907135 36534810

