# Supplementary material for: De novo mapping of α-helix recognition sites on protein surfaces using unbiased libraries
Source: Proc Natl Acad Sci U S A. 2022 Dec 19;119(52):e2210435119. doi: 10.1073/pnas.2210435119 (PMC9907135; doi:10.1073/pnas.2210435119)
Supplement: Supplementary file 1 — Appendix 01 (PDF) [file pnas.2210435119.sapp.pdf]

PNAS

Supplementary Information for

De novo epitope mapping of α-helix recognition sites on protein surfaces using unbiased libraries

Kunhua Li, Olena S. Tokareva, Ty M. Thomson, Sebastian C.T. Wahl, Tara L. Travaline, Jessica D. Ramirez, Santosh K. Choudary, Sorabh Agarwal, Ward G. Walkup IV, Tivoli J. Olsen, Matthew J. Brennan, Gregory L. Verdine, John H. McGee

Corresponding authors: Gregory L. Verdine and John H. McGee
Email: gregory_verdine@harvard.edu, jmcgee@fogpharma.com

This PDF file includes:

SI Materials and Methods
Figures S1 to S7
Tables S1 to S2

Legends for Datasets S1 to S2

SI References

Other supplementary materials for this manuscript include the following:

Datasets S1 to S2

Supporting Information Materials and Methods

1 Crosslinker Synthesis

N,N'-(1,4-phenylene)bis(2-bromoacetamide) was synthesized by adding bromoacetyl bromide (1.1 mmol) to a solution of p-phenylenediamine (0.5 mmol) in dichloromethane (DCM) (5 mL), dry pyridine (1.2 mmol), and 4-(dimethylamino)pyridine (13 µmol) at 0°C. The mixture was stirred at 25°C for 3 hours. The reaction mixture was filtered and washed with DCM to obtain N,N'-(1,4-phenylene)bis(2-bromoacetamide) as an off-white solid. ^{1}H NMR (DMF-d7, 500 MHz): δ 4.12 (s, 4H), δ 7.67 (s, 4H), δ 10.47 (s, 2H).

2 Helicon Synthesis and Cysteine Bisalkylation

Helicons were synthesized at 100 µmol scale on Rink Amide resin (~0.5 mmol/g) using standard Fmoc-based solid phase peptide synthesis workflows. Specifically, the Fmoc-protected resin was swollen using N,N-dimethylformamide (DMF) before coupling the first amino acid. Fmoc-deprotection was performed by treating the resin with 20% (v/v) piperidine in DMF. The amino acid coupling was performed in DMF with 4 equivalents of Fmoc-protected amino acid, 8 equivalents of ethyl cyanohydroxyiminoacetate (oxyma), and 4 equivalents of N,N'-diisopropylcarbodiimide (DIC). The peptides were globally deprotected and cleaved off the resin by treating the resin with a cleavage cocktail composed of 92.5% (v/v) trifluoroacetic acid (TFA), 2.5% (v/v) water, 2.5% (v/v) triisopropylsilane, and 2.5% (v/v) mercaptopropionic acid for 2 hours. The crude peptide was precipitated by adding ice-cold isopropyl ether to the concentrated cleavage cocktail. The precipitated peptide was pelleted by centrifugation and dried under nitrogen.

The crude peptide was dissolved in DMSO before proceeding with the cysteine bisalkylation/stapling reaction. The DMSO stock was diluted in a 2:1 solvent mixture of acetonitrile and 50 mM ammonium hydroxide. The pH of the solution was adjusted to ~8.5 using N,N-Diisopropylethylamine (DIPEA). 1.3 equivalents of the alkylating agent, N,N'-(1,4-phenylene)bis(2-bromoacetamide) in DMF were added to the crude peptide solution. The reaction mixture was stirred at room temperature for at least two hours or until the reaction had been completed. The progress of the reaction was monitored by analytical HPLC and mass spectrometry. The final reaction was quenched by β-mercaptoethanol before lyophilizing the reaction mixture. The lyophilized reaction mixture was dissolved in DMSO for purification.

The crude peptide mixtures were purified by preparatory HPLC (solvent A: water with 0.1% (v/v) FA; solvent B: acetonitrile with 0.1% (v/v) FA) using a C18 column. Analytical HPLC and mass spectrometry were used to characterize the peptides. The observed masses of the final peptide products are summarized in Table S3.

3 Circular Dichroism (CD) Spectroscopy

CD spectra of stapled and unstapled peptide pairs were obtained using Aviv Biomedical, Inc. 420 CD spectrometer with peptide concentrations at 25 µM or 50 µM in 20 mM phosphate buffer at pH=7.4. The CD measurements were obtained using a cuvette with a 1 mm pathlength at a fixed temperature of 25°C. Three scans were obtained at every 1-nm interval in the wavelengths ranging from 190 to 260 nm. The buffer background was subtracted from each CD spectrum, followed by smoothing the curves by the moving-means method with a convolution width of 2 data points. Next, the smoothed baseline between 250-260 nm was subtracted from the smoothed spectrum of the sample. Finally, the CD measurements were converted to mean residue molar ellipticity ($deg \bullet cm^2 \bullet dmol^{-1}$) for data visualization (1). As DPA residues were used as an N-terminal nonhelical cap, they were ignored for the mean residue molar ellipticity calculations. The percent helicity of peptides was calculated using the ratio of $[\theta]_{222}/[\theta]_{max}$. $[\theta]_{max}$ for the peptides was estimated to be -26964.28 using the formula below (2, 3).

$$[\theta]_{max} = (-44000 + 250T)\left(1 - \frac{k}{n}\right)$$
$$T = 25°C, k = 4, n = 14$$

4 Phage Library construction (primers, protocol, crosslinking, and DNA sequencing)

Phage-displayed peptide libraries were constructed using the filamentous bacteriophage vector M13KE (New England Biolabs, Ipswich, MA). We recommend closely following the protocol guidelines in the New England Biolabs Ph.D.™ Peptide Display Cloning System kit. Briefly, library of oligonucleotides were chemically synthesized using a mix of trimer phosphoramides (Glen Research, Sterling, VA) lacking cysteine, lysine, proline, and glycine, then annealed, extended, and ligated into a digested M13KE vector. The sense strand contains the library sequence, 5'-CATGCCCGGGTACCTTTCTATTCTCACTCTGCGGATCCGGCGXXXTGCXXGCAGCAXXTGTXXXGGTGGTTCTGGCTGGGGTCGTGGTTC-3', where X represents a single trimer phosphoramide incorporation, flanked by KpnI site. The antisense strand complements the 3' end of the sense strand to allow Klenow extension, 5'-CATGTTTCGGCCGAACCACGACCTGCGCCAGAACCAC-3'. The antisense strand possesses an EagI site. Annealed library inserts, along with the M13KE vector, were digested with EagI and KpnI for 5 hours, and the digested products were purified using Monarch PCR and DNA cleanup kit (New England Biolabs, Ipswich, MA), followed by T4 ligation (New England Biolabs, Ipswich, MA). The resulting library-containing vector was transformed into *E. coli* strain ER2738 (Lucigen, Middleton, WI) by electroporation, reserving and plating post-rescue to determine library diversity (4). Between 5 x 10^7 and 1 x 10^8 individual phage clones (established by a traditional plaque assay) were used for library amplification. Phages were amplified by adding the post-rescue electroporated cells to a robustly growing E. coli culture at early-log phase for 5 h in 500 mL LB media supplemented with 100 µM each of $MgCl_2$ and $CaCl_2$, with shaking at 37˚C. *E. coli* cells were pelleting at 5000 x g and supernatant was removed. Phage particles were collected from the supernatant by addition of 1/5 volume of 20% (w/v) polyethylene glycol 8000, 2.5 M NaCl, followed by overnight incubation at 4˚C, pelleted at 5000 x g, and resuspended in Tris-buffered saline (TBS). Phage-displayed Helicon libraries were further purified by repeating the precipitation, pelleting, and resuspension steps. After the final resuspension step, a plaque assay was performed to assess the overall titer of the phage display library. We usually observe

between 5 x 10^{12} and 1 x 10^{13} phage particles in each amplified library. Phage-displayed Helicon libraries were covalently crosslinked (stapled) by diluting the phage particle solution in TBS to an OD_{600} of 1.0 and adding dithiothreitol to a concentration of 1 mM, followed by dialysis against 100 volumes of 20 mM NH_4CO_3, 2 mM EDTA, pH ~8 for 30-60 min, followed by addition of the dialyzed phage to a solution of crosslinker prepared in 20 mM NH_4CO_3, 2 mM EDTA, pH ~8 (final crosslinker concentration is 200 µM. As the crosslinker does not completely dissolve in buffer, we briefly sonicated the solution immediately prior to mixing with phage to disperse the solid into a fine suspension) and incubation with rotation for 2 hours at 32˚C. Excess crosslinker was removed first by pelleting at 5000 x g and decanting, followed by addition of dithiothreitol to a concentration of 0.25 mM with incubation for 10 minutes, and then addition of iodoacetamide to a concentration of 0.75 mM with incubation for a further 10 minutes. Ellman's reagent (5,5′-dithiobis-(2-nitrobenzoic acid) was used to track the quenching of DTT until all thiols were capped. Phage particles were further purified by repeating the precipitation, pelleting, and resuspension steps described above for purification from *E. coli* culture, then are stored as solutions in 50% v/v glycerol in TBS at -80˚C at >10^{12} pfu/mL. Next-generation sequencing was performed to assess the library quality (details can be found in the Phage NGS section). We typically sequenced between 10^6 – 10^7 phage particles and found that on average, between 95%-98% of all reads have the correct library structure. Mass spectrometry analysis of crosslinked phage was performed by adding 15 µL of phage samples to 2.5 µL of a solution of Trypsin at 1.0 mg/ml freshly prepared in 5 mM acetic acid and then pH adjusted by mixing 1:1 with 100 mM Tris pH 8. After 1 hour of digestion at room temperature, each cleavage reaction was quenched with 15 µL of 20% ACN + 1% formic acid, and analyzed a Q-Exactive Plus mass spectrometer equipped with an Ultimate 3000 LC system (Thermo Electron) and a Aeris™ C18 column (Phenomenex). Finally, individual phage library members were characterized by DNA sequencing. Well-separated blue plaques were picked from the LB/IPTG/Xgal Agar plates in 50 µL of water. 2 µL of resuspended template was mixed with 23 µL of the amplification master mix containing OneTaq DNA polymers (NEB, Ipswitch, MA) and two 10 µM M13KE sequence-specific amplification primers (NEB, Ipswitch, MA). Routine PCR was performed, and samples were submitted for standard Sanger sequencing (GENEWIZ, Cambridge, MA). Prior to library screening, we performed and recommend deep sequencing (as described below) of the library to ensure that it is high in sequence diversity and is not dominated by a small number of individual sequences.

5 Phage Library Screening

Phage screening was performed using biotinylated proteins bound to streptavidin magnetic beads (Dynabeads MyOne Streptavidin T1, Thermo Fisher Scientific, Waltham, MA). 10^{10} phage particles were added to each phage screening sample, to ensure approximately 100 copies of each of the 10^8 library members. Phage display libraries were incubated with streptavidin magnetic beads for 1 hour at room temperature in a buffer made of 1X TBS, 1 mM $MgCl_2$, 1% w/v BSA, 0.1% Tween-20, 0.02% w/v sodium azide, 5% w/v nonfat milk to remove bead-binding library members. Briefly, Dynabeads were prepared in a 15ml Falcon tube according to a manufacturing protocol from Thermo Fisher Scientific, diluted phage display libraries were added to the magnetic beads. After an incubation period, the tube was placed on a magnet for 1 min to separate bead-bound and non-bead-bound phage library members. Supernatant containing bead

depleting phage library was collected and beads were discarded. For each screening condition, 100 µL of 2 µM biotinylated protein was captured with 0.5 mg of streptavidin-coated magnetic beads that have been previously blocked with 1% BSA, 0.1% Tween, 2% glycerol in 1x TBS pH 7.4 at room temperature for 15 minutes in 96-well plates, followed by removal of the supernatant using a plate magnet and prompt but gentle resuspension of the beads in 50 µL of the same buffer. Next, 150 µL of the depleted phage library was added to each well for 200 µL final volume, the plates were sealed, and the screening reactions incubated at room temperature for 45 minutes, with rotation to maintain beads in solution. We inspected these solutions to confirm that beads had not aggregated or crashed out of solution, which can be indicative of protein aggregation. Following binding, beads were washed 5x with ice-cold 1x TBS, 1 mM $MgCl_2$, 1% w/v BSA, 0.1% Tween-20, 0.02% w/v sodium azide, 2% w/v glycerol. Washing steps can be performed, as in our case, with an automated bead handler such as a KingFisher (Thermo Fisher) or manually. If washing beads manually for a screen of 48 wells or fewer, we recommend working quickly to ensure that washing occurs consistently between samples and occurs in 20 minutes or less. Given the speed required to complete all steps, manual washing of greater than 48 samples is not recommended. Target-bound phage library members are directly processed for NGS.

6 Phage Next-Generation Sequencing (NGS)

This protocol is used to perform NGS for newly built phage display libraries and to identify target-bound phage library members after a phage screen. To sequence the phage-displayed peptide library members, phage particles are removed from the beads by a denaturation step at 95˚C for 15 min in 25 mM Tris pH 8, 50 mM NaCl, 0.5% Tween-20. Prior to boiling, 10,000 copies of a phage clone of known sequence (not a library member) are spiked in to each well to enable cross-well normalization of sequence reads. The sequence of the spike-in clone is TCTCACTCTGCGCCGGAATGCATTCTGGATTGCCATGTGGCGCGCGTGTGGGGTGGTTCT. A two-step low-cycled PCR is performed to introduce Illumina adaptors and 10bp TruSeq DNA UD Indexes (Illumina, San Diego, CA) to the 3' and 5' ends of amplicons with M13KE Forward and M13KE Reverse primers (M13KE Forward: 5'-TCGTCGGCAGCGTCAGATGTGTATAAGAGACAGTTCGCAATTCCTTTAGTGG-3' and M13KE Reverse: 5'-GTCTCGTGGGCTCGGAGATGTGTATAAGAGACAGATTTTCTGTATGGGATTTTGCTAA-3') similar to Illumina's 16S Metagenomic Sequencing Library Preparation protocol. The NGS libraries are sequenced by an Illumina NovaSeq platform using a 2x150-bp high-output kit (Illumina, San Diego, CA).

7 Hit ID and Clustering

NGS reads were trimmed for quality (Phred score ≥ 18) and filtered for sequences that matched the design of the phage library (Fig 2A). Counts for each unique sequence were tallied, and then normalized by the counts of the spike-in sequence added to each sample. A metric called Hit Strength was computed for each sequence as the fold change between the normalized counts in the highest target concentration sample and the normalized counts in the blank bead samples (averaged across experimental replicates). When 0 counts were observed for a sequence in blank bead samples, a count of 0.5 was used to prevent dividing by zero. Sequences with a hit strength greater than 5 were then subjected to hierarchical clustering to identify sequence families. Pairwise distances between sequences *i* and *j* were computed using

$$distance_{ij} = \left(1 - \frac{score_{ij}}{score_{ii}}\right)\left(1 - \frac{score_{ij}}{score_{jj}}\right),$$

where $score_{ij}$ is the alignment scores based on a modified BLOSUM62 substitution matrix. We decreased the tryptophan-tryptophan match score from 11 to 7 in the BLOSUM62 substitution matrix to prevent overly biasing clustering towards tryptophans. Hierarchical clustering using average linkage was used to group the sequences into families. To avoid clustering using a large number of sequences, which is computationally intensive and can make it difficult to identify small clusters, we performed multiple rounds of clustering. First, we sorted sequences by descending hit strength. We then took the top 1000 (first round of clustering), top 2000 (2nd round of clustering), or top 3000 ($\geq 3^{rd}$ round of clustering) sequences, and subjected them to clustering as described above. Clusters of sequences with high sequence similarity (sequence "families") were identified at each round and removed from the pool of sequence for subsequent rounds. Sequences subjected to three rounds of clustering without falling into a sequence family were similarly dropped from subsequent rounds of clustering under the assumption that they did not belong to a sequence family. The process was halted after 10 rounds of clustering, or when no sequences remained in the list.

8 β-Catenin Surface Plasmon Resonance (SPR)

SPR experiments were performed on a Biacore™ 8K (Cytiva) instrument at 25°C. Test peptides were diluted into running buffer (50 mM Tris pH 8.0, 300 mM NaCl, 2% glycerol, 0.5 mM TCEP, 0.5 mM EDTA, 0.005% Tween-20, 1% DMSO). Compounds were diluted to 10 µM or 1 µM and serially diluted 1:3 for seven concentrations and two blanks (7-point three-fold peptide dilution series with top concentration = 10 µM). Biotinylated β-catenin residues 134-665 (Uniprot ID P35222) was immobilized to the active surface of the sensor chip for 25 seconds at 10 µL/min using the Biotin CAPture Kit, Series S (Cytiva) and compounds were injected over the reference and active surfaces for 180 seconds at 65 µL/min then allowed to dissociate for 400 seconds. Results were analyzed using the Biacore™ Insight Evaluation software, with double-referencing and fitted to a 1:1 binding affinity model.

9 β-catenin-TCF Competition by Fluorescence Polarization

Compounds at 10 mM in DMSO were serially diluted 1:3 in DMSO for a total of 11 concentrations using a Mosquito LV (SPT Labtech), then diluted 1000-fold in buffer (50 mM HEPES, pH 7.5, 125 mM NaCl, 2% glycerol, 0.5 mM EDTA, 0.05% v/v pluronic acid) in duplicate by the Mosquito LV (SPT Labtech) into a black polystyrene 384-well plate (Corning) (11-point three-fold peptide dilution series with top concentration = 10 µM) Probe solution (10 nM full-length β-catenin (Uniprot ID P35222), mixed with 10 nM 5FAM-labeled TCF4 residues 10-53 (Uniprot ID Q9NQB0) peptide (FP04872) in buffer) was prepared and plated using the MultiDrop Combi (Thermo Fisher) for a total reaction pool of 40 µL. The plate was incubated and protected from light for 1 hour at room temperature prior to read. Reads were performed on a CLARIOstar plate reader (BMG Labtech) with excitation at 485 nm, emission at 525 nm,

and cutoff at 504 nm. Data were fitted to a 1:1 binding model with Hill slope using an in-house script.

10 β-catenin-Axin Competition by Fluorescence Polarization

Compounds at 10 mM in DMSO were serially diluted 1:3 in DMSO for a total of 11 concentrations using the Mosquito LV (SPT Labtech), then diluted 1000-fold in buffer (50 mM HEPES, pH 7.5, 125 mM NaCl, 2% glycerol, 0.5 mM EDTA, 0.05% v/v pluronic acid) in duplicate by the Mosquito LV (SPT Labtech) into a black polystyrene 384-well plate (Corning). Probe solution (15 nM full-length β-catenin (Uniprot ID P35222), mixed with 20nM FITC labeled fStAx-33 (5) peptide (FP00013) in buffer) was prepared and plated using the MultiDrop Combi (Thermo Fisher) for a total reaction pool of 40 μL. The plate was incubated protected from light for 1 hour at room temperature prior to read. Reads were performed on a CLARIOstar plate reader (BMG Labtech) with excitation at 485 nm, emission at 525 nm, and cutoff at 504 nm. Data were fitted to a 1:1 binding model with Hill slope using an in-house script.

11 Measurement of the Cell Association of Helicons

A "Source" plate of 47 test compounds was prepared at a concentration of 1mM in 90% DMSO in a 500 µL 96-well plate (2 replicates for each compound with 2 DMSO blanks). The 96-well format with one compound per well was maintained for all transfers throughout the protocol. A 2000 µL 96-well v-bottom plate ("Cells" plate) was used to dilute 1.25 µL of compounds from the Source plate into 500 µL of Expi293™ Expression Medium. Each well also received 500 µL of Expi293™ cells (Thermo Fisher) for a total concentration of $1x10^6$ cells/mL in 1 mL of Expi293™ Expression Medium. Baseline cell health at the time of compound addition was measured using CellTiter-Glo™ 2.0 Reagent (CTG) and a GloMax Discover reader (Promega). The Cells plate was incubated in an Infors HT Multitron Pro shaking incubator at 1000rpm, 37°C, 8.0% CO_2, ~55% humidity, for 22 hours, along with four plates of DI water to maintain humidity. After 22 hours, the cells were sampled again for post-incubation CTG analysis of cell health. CellTiter-Glo fold-change is calculated as the luminescence readout at T_{final} divided by the luminescence readout at T_{inital} (time at which peptide was added). The cells were washed twice with 200 µL of Dulbecco's Phosphate Buffered Saline (DPBS) and transferred to a 500 µL 96-well "Final Assay" plate. After the second wash, the cells were resuspended in 80 µL of buffer (90% DPBS, 10% dimethyl sulfoxide (DMSO), 10 µM of a mixture of nonstapled 14-mer peptides with randomized sequences) in the Final Assay plate. Cell lysis was induced by the addition of 240 µL of ammonium hydroxide and 2 hours of shaking at 37°C. All solvents were removed via 23 hours in a SpeedVac vacuum concentrator (Thermo Fisher). The dried compounds and cell debris were resuspended using 180 µL of resuspension buffer (47.5% Acetonitrile (ACN) with 0.1% formic acid (FA), 47.5% H_2O with 0.1% FA, 5% DMSO) and shaking for 3 hours at 600 rpm. Once resuspended, the cell debris was separated from the resuspended compounds via centrifugation at 3220 rcf for 20 min. A portion of compound-containing supernatant from each well was transferred to a corresponding well in a 384-well plate for mass spectrometry analysis. Matching "cell-free" wells for all "cells" wells were plated in the same 384-well plate. The "cell-free" wells were prepared by adding 1 µL of 0.1 mM compounds from the Source plate to 19 µL of input buffer (47.5% ACN with 0.1% FA, 47.5% H_2O with 0.1% FA, 5% DMSO, 10 µM of a

mixture of nonstapled 14-mer peptides with randomized sequences), then adding 2 µL of this 5 µM-dilution to 58 µL of input buffer in the 384-well mass spectrometry plate. All samples were analyzed using mass spectrometry. The percentage of compound in cells after treatment and wash was computed as the percentage of the total compound added to the Cells samples that was present in the cell fraction after removal of the extracellular media. Compounds were quantitated by mass spectrometry, and the signal in the cell-free samples were used as a single-point calibration curve to convert from signal intensity in the Cells sample to the percentage of the total amount added.

12 β-catenin protein production

β-catenin protein (residues 134-665) with a N-terminal His6-yBBr-TEV tag was recombinantly expressed in E. coli BL21 (DE3) pLysS cells (Thermo Fisher) from pET28a vectors (Novagen). The cells were induced at OD_{600}=0.6 with 0.15 mM isopropyl β-D-1-thiogalactopyranoside (IPTG) for 16 hours at 16°C, then harvested and resuspended in 25 mM Tris pH 8.0, 200 mM NaCl, 10% glycerol, 20 mM imidazole, 1 mM PMSF. For purification, the pellet was lysed with a tip sonicator, pelleted at 22,000 x g for 30 minutes at 4°C, then the supernatant was purified with HisTrap HP columns (Cytiva), eluting with 250 mM imidazole. For crystallography efforts, protein was TEV-cleaved by adding TEV protease at a ratio of 1:10 protease to protein and incubated overnight at 4°C. For biochemical assays, protein was biotinylated via the yBBr reaction according to standard procedures (6). All proteins were concentrated using Amicon spin filters (Millipore Sigma) then diluted into 25 mM Tris, pH 8.8, 1 mM DTT, 10% glycerol and loaded onto a Q HP (Cytiva) column. Proteins were eluted with a gradient from 50 mM to 600 mM NaCl. Protein-containing fractions were pooled, concentrated and loaded onto a Superdex® 10/300 200pg (Cytiva) SEC column. Purified proteins were eluted isocratically in 25 mM Tris-HCl, pH 8.8, 10% glycerol, 300 mM NaCl and fractions containing pure protein were collected and pooled.

13 RNF31 Protein production (UBA, PUB, Sharpin)

UBA Domain

RNF31 UBA domain protein (residues 480-639) with N-terminal Thioredoxin-TEV-6xhis-yBBr-3C tags was recombinantly expressed in E. coli BL21 (DE3) pLysS cells (Thermo Fisher) from pET-derived expression vectors (Novagen). The cells were induced at OD_{600}=0.6 with 0.25 mM IPTG for 16 hours at 16°C, then harvested and resuspended in 50 mM HEPES pH 7.5, 500 mM NaCl, 15 mM imidazole, 1 mM TCEP, 2 mM ATP, 10 mM $MgCl_2$, 0.1x BugBuster®, 5% glycerol 25 U/mL Ready-Lyse™, 25 U/mL Omnicleave™, and 1 tablet Roche cOmplete™ EDTA-free per 50 mL. For purification, the pellet was lysed with a tip sonicator, pelleted at 22,000 x g for 40 minutes at 4°C, then the supernatant was purified with HisTrap HP columns (Cytiva), eluting with 500 mM imidazole. For crystallography efforts, protein was cleaved by adding 3C protease at a ratio of 1:40 protease to protein and incubating overnight at 4°C. For biochemical experiments, protein was cleaved by adding TEV protease at a ratio of 1:10 protease to protein and incubating overnight at 4°C overnight. TEV cleaved proteins were then biotinylated via the yBBr reaction. Proteins were concentrated and injected over a Superdex® HiLoad 16/600 75pg SEC column pre-equilibrated with 50 mM HEPES pH 7.5, 150 mM NaCl, 1 mM TCEP and 5% Glycerol.

Proteins were eluted isocratically and fractions containing pure protein were collected and pooled.

PUB domain

RNF31 PUB domain protein (residues 1-179) with N-terminal MBP-TEV-6xHis-YBBR-3C tags was recombinantly expressed in E. coli BL21 (DE3) pLysS cells (Thermo Fisher) from pET-derived expression vectors (Novagen). The cells were induced at OD_{600}=0.6 with 0.15 mM IPTG for 16 hours at 16°C, then harvested and resuspended in 50 mM HEPES pH 7.5, 500 mM NaCl, 1 mM TCEP, 2 mM ATP, 5 mM $MgCl_2$, 5% Glycerol, and 1 mM PMSF. For purification, the pellet was lysed with a tip sonicator, pelleted at 22,000 x g for 30 minutes at 4°C, then the supernatant was purified with Ni-NTA resin (Qiagen), eluting with 250 mM imidazole. For crystallography efforts, protein was cleaved by adding 3C protease at a ratio of 1:40 protease to protein and incubating overnight at 4°C. For biochemical experiments, protein was cleaved by adding TEV protease at a ratio of 1:10 protease to protein and incubating overnight at 4°C overnight. TEV cleaved proteins were then biotinylated via the yBBr reaction. Proteins were concentrated and injected over a Superdex® HiLoad 16/600 75pg SEC column pre-equilibrated with 25 mM HEPES pH 7.5, 150 mM NaCl, 1 mM TCEP. Proteins were eluted isocratically and fractions containing pure protein were collected and pooled.

Sharpin

Sharpin ubiquitin-like domain protein (residues 206-309) with N-terminal Thioredoxin-6xHis-Thrombin-yBBr-TEV tags was recombinantly expressed in E. coli BL21 (DE3) pLysS cells (Thermo Fisher) from pET-derived expression vectors (Novagen). The cells were induced at OD_{600}=0.6 with 0.2 mM IPTG for 16 hours at 16°C, then harvested and resuspended in 50 mM HEPES pH 7.5, 500 mM NaCl, 15 mM imidazole, 1 mM TCEP, 2 mM ATP, 10 mM MgCl2, 0.1x BugBuster®, 5% glycerol 25 U/mL Ready-Lyse™, 25 U/mL Omnicleave™, and 1 tablet Roche Complete EDTA-free per 50 mL. For purification, the pellet was lysed with a tip sonicator, pelleted at 22,000 x g for 30 minutes at 4°C, then the supernatant was purified with HisTrap HP columns (Cytiva), eluting with 500 mM imidazole. For biochemical experiments, protein was cleaved by adding TEV protease at a ratio of 1:10 protease to protein and incubating overnight at 4°C overnight. TEV cleaved proteins were then biotinylated via the yBBr reaction. Proteins were concentrated and injected over a Superdex® HiLoad 16/600 75pg SEC column pre-equilibrated with 50 mM HEPES pH 7.5, 150 mM NaCl, 1 mM TCEP and 5% Glycerol. Proteins were eluted isocratically and fractions containing pure protein were collected and pooled.

14 RNF31 UBA and PUB SPR

All SPR experiments were performed on a Biacore 8K (Cytiva) instrument at 25°C. For kinetics experiments, the instrument was primed with 10 mM HEPES, pH7.5, 150 mM NaCl, 0.05% Tween 20, 1% DMSO. A CAP Series S sensor chip was docked and pre-conditioned with 3 injections of 1X CAP regeneration solution to remove unbound capture reagent from the surface. Biotinylated RNF31 UBA and PUB domain proteins were diluted to 1 μM in running buffer. FP06655 was diluted to 1 μM in running buffer and serially diluted 1:3 for a total of 8 concentrations and a blank (8-point four-fold peptide dilution series with top concentration = 1 μM.). Otulin and test peptides were diluted to 10 μM in running buffer and serially diluted 1:3

for a total of 8 concentrations and a blank. Proteins were captured to the active surface of the sensor chip for 60 seconds at 5 μL/min and the peptides were injected over the reference and active surfaces for 180 seconds at 50 μL/min then allowed to dissociate for 360 seconds. Surface was regenerated with a 120-second injection of CAP regeneration solution each cycle. Data was analyzed using Biacore Insight Evaluation software (Cytiva). Sensorgrams were double-referenced and fit to 1:1 binding affinity model.

15 RNF31-Otulin competition

Fluorescence Polarization

RNF31 PUB domain was diluted to 1.6 μM in assay buffer (10 mM HEPES pH 7.4, 150 mM NaCl, 0.05% Tween 20) and pipetted into a 384-well black microplate (Corning) in a final volume of 20 μL. Test peptides were added to the plate (40 nL each) serially diluted 3-fold from 10 mM and the plate was incubated at room temperature for 20 minutes (11-point three-fold peptide dilution series with top concentration = 10 μM). FITC-labeled Otulin peptide (residues 49-67) (FP16923) was diluted to 40 nM in assay buffer, then 20 μL of the stock was added to the plate for a final volume of 40 μL. The plate was incubated for 60 minutes at room temperature, then fluorescence anisotropy was recorded on a CLARIOstar (BMG LabTech) with excitation at 485 nm, emission at 525 nm, and cutoff at 515 nm. Data were plotted using Prism (Graphpad) and fit to a one-site specific binding model with Hill coefficient.

SPR ABA Competition

All SPR experiments were performed on a Biacore 8K (Cytiva) instrument at 25°C. For kinetics experiments, the instrument was primed with 10 mM HEPES, pH 7.5, 150 mM NaCl, 0.05% Tween 20, 1% DMSO. A SA Series S sensor chip was docked and pre-conditioned with three injections of 50mM NaOH/1M NaCl to remove unbound streptavidin from the surface. Biotinylated RNF31 PUB domains were diluted to 2 μM in running buffer. FP06649 and FP06652 were diluted to 10 μM in running buffer. Otulin peptide (residues 49-67) was diluted to 10 μM in running buffer. Proteins were captured on the active surface of the sensor chip for 300 seconds at 1 μL/min. For each injection, compounds were injected over the surface for 120 seconds at 30 μL/min to achieve equilibrium binding. Otulin was then injected for 60 seconds at 30 μL/min in the absence or presence of competing compound over the surface. Surface was regenerated with an injection of 1M sodium chloride after each cycle. Data was analyzed using Biacore Insight Evaluation software (Cytiva). Sensorgrams were double-referenced and evaluated for competition.

16 RNF31-Sharpin competition by fluorescence polarization

RNF31 UBA domain, and FAM-labeled RNF31-binding peptide, (FP12122), were diluted to 400 nM and 40 nM, respectively in assay buffer (10 mM HEPES pH 7.4, 150 mM NaCl, 0.05% Tween 20) and pipetted into a 384-well black microplate (Corning) in a final volume of 20 μL. Recombinant Sharpin/SIPL1 UBL protein (residues 153-256), as well as selected control peptides, were added to the plate (20 μL each), serially diluted 3-fold from 10 μM (10-point three-fold peptide or Sharpin dilution series with top concentration = 3.3 μM). The plate was incubated for 60 minutes at room temperature, then fluorescence anisotropy was recorded on

a CLARIOstar (BMG LabTech) with excitation at 485 nm, emission at 525 nm, and cutoff at 515 nm. Data were plotted using Prism (Graphpad) and fit to a one-site specific binding model with Hill coefficient.

17 CDK2 and PPIA protein production

CDK2

Full length CDK2 (residues 1-298) with an N-terminal GST-3C-6xhis-TEV tag was recombinantly expressed in Sf9 cells according to the Bac-to-Bac protocol (Thermo Fisher). Briefly, Sf9 cells were plated at $1x10^6$ cells in 2mL Sf-900™ II media (Thermo Fisher) into a 6-well cell culture plate. Cells were transfected with purified bacmid diluted in OptiMem™ media using Cellfectin™ reagent. Cells were incubated at 27°C for 5 days. Cells and supernatant were removed from plate and centrifuged. P1 virus was collected and cell pellet was evaluated for protein expression by Western blot. P2 virus was generated by infecting $2x10^6$ cells/mL Sf9 in 50 mL Sf-900™ II media with 500 mL P1 virus. Cells were incubated with shaking at 27°C for 5 days. Cells were centrifuged at 1500 rpm at room temperature for 5 minutes. Supernatant was stored at 4°C as P2 virus stock and pellet was evaluated by Western blot for protein expression. Protein was expressed by seeding Sf9 cells at $2x10^6$ cells/mL in Sf-900™ II media and infecting at an MOI of 1:200. Cultures were incubated with shaking for 72 hours at 27°C. Cells were harvested and supernatant was discarded. Pellets were resuspended in 25mM HEPES, pH 7.5, 300 NaCl, 10% glycerol, 0.5 mM PMSF and then sonicated with a tip sonicator. Lysates were centrifuged at 22,000 x g for 30 minutes at 4°C. Clarified lysate was purified with a GSTrap™ (Cytiva) pre-equilibrated in 25 mM HEPES, pH 7.5, 300 mM NaCl, 10% glycerol. Protein was eluted with 25 mM HEPES, pH 7.5, 300 mM NaCl, 10% glycerol, 10 mM GSH. Eluted protein was cleaved by combining protein with TEV protease at a ratio of 1:10 protease to protein and incubating at room temperature for 40 hours. Cleaved protein was then dialyzed into 25 mM HEPES, pH 7.5, 300 mM NaCl, 10% glycerol and re-injected over a GSTrap™ to remove cleaved tags. Purified protein was concentrated and centrifuged at 22,000 x g for 10 minutes at 4°C to remove soluble aggregates. Protein was then loaded onto a Superdex™ HiLoad 16/600 75pg SEC column pre-equilibrated with 20 mM HEPES, pH 7.5, 150 mM NaCl, 2% glycerol, 2 mM DTT. Protein was eluted isocratically at 0.5 mL/min. Finally, protein was centrifuged at 22,000 x g for 10 minutes at 4°C to remove soluble aggregates, then aliquoted and frozen.

CDK2 (pT160)

CDK2 with T160 phosphorylation was obtained through the co-expression of GST-3C-6xhis-TEV-CDK2 above with *Saccharomyces cerevisiae* GST–Cak1. The phosphorylation of pT160 was confirmed with phospho-CDK2 (Thr160) antibody (Cell Signaling #2561) and mass spectrometry.

CDK2 (pT160)/CCNE1

GST-CCNE1 (residues 81–363) was expressed in E. coli BL21 (DE3) pLys S cells. Briefly, cells were grown at 37°C to OD_{600} reached 0.8 and induced with 0.1 mM IPTG overnight at 20°C. Cells were lysed in 10 mM HEPES, 150 mM NaCl, pH 7.5 and pelleted with centrifugation. The cell lysate was incubated with glutathione–Sepharose 4B beads with purified CDK2 harboring pT160. After elution with 20 mM glutathione, pCDK2/GST-cyclin E1 was digested with GST-3C protease overnight. The pCDK2/cyclin E1 complex was collected and loaded onto a Superdex™

16/600 75pg (Cytiva) SEC column pre-equilibrated with 20mM Tris pH 7.4, 200mM NaCl, 10% glycerol, 1.0mM DTT. Purified proteins were eluted isocratically. Protein fractions were collected, concentrated, aliquoted and frozen.

PPIA

Full length PPIA (residues 1-165) with an N-terminal 6xhis-yBBR-TEV tag was recombinantly expressed in E. coli BL21 (DE3) CodonPlus RIPL cells (Agilent) from pET-derived expression vectors (Novagen). The cells were induced at OD_{600}=0.6 with 0.15 mM isopropyl β-D-1-thiogalactopyranoside (IPTG) for 16 hours at 16°C, then harvested and resuspended in PBS pH 7.4 with 1mM PMSF. For purification, the pellet was lysed with a tip sonicator, pelleted at 22,000 x g for 30 minutes at 4°C. Supernatant was collected then centrifuged again at 22,000 x g for 30 minutes at 4°C. The supernatant was purified with Ni-NTA resin (Qiagen), eluting with 250 mM imidazole. Protein was TEV cleaved by adding TEV protease at a ratio of 1:10 protease to protein and incubated for 4 hours at 4°C. Protein was then concentrated and diluted into 20 mM HEPES pH 7.0, 5% glycerol and centrifuged at 22,000 x g for 10 min at 4°C. The supernatant was loaded onto a SP HP (Cytiva) column pre-equilibrated with 20 mM HEPES pH 7.0, 5% glycerol. Purified protein was eluted with a gradient from 0 mM to 1 mM NaCl. Protein fractions were pooled, concentrated then centrifuged at 22,000 x g for 10 min at 4°C. Supernatant was collected and loaded onto a Superdex™ 16/600 75pg (Cytiva) SEC column pre-equilibrated with PBS pH 7.4. Purified proteins were eluted isocratically in PBS pH 7.4. Protein fractions were collected, concentrated, aliquoted and frozen.

18 SPR for CDK2 and the CDK2/CCNE1 complex

SPR experiments were performed on a Biacore S200 or 8K (Cytiva) instrument at 25°C in 1x HBS-P+ buffer (Cytiva) with 1% DMSO. A SA Series S sensor chip was docked and pre-conditioned with three injections of 50 mM NaOH/1 M NaCl to remove unbound streptavidin from the surface. CDK2 protein, or CDK2: CCNE1 complex, was diluted to 5 µg/mL in running buffer and immobilized to channels 1 through 8 at 5 µL/min for 50-80 seconds for a final immobilization level of ~1800 RU. Peptides were diluted to 5 µM in running buffer then serially diluted 2-fold for a total of seven concentrations with one blank (7-point two-fold peptide dilution series with top concentration = 5 µM.). Compounds were injected over the immobilized and reference surfaces at 30 µL/min for 60 seconds and then allowed to dissociate for 180 seconds without surface regeneration. Sensorgrams were double-referenced and fit to a 1:1 steady state affinity model.

19 ATP competition of CDK2

For ATP competition experiments, 50 µM of selected CDK2-binding compounds were added into 20 nM Bodipy-ATP- γS (Thermo Fisher) and 2 µM CDK2 in an assay buffer contained 20 mM Tris pH 8, 300 mM NaCl, 10% (v/v) glycerol, 2 mM TCEP, and 10 mM $MgCl_2$, and pipetted into a 384-well black microplate (Corning) in a final volume of 40 µL. The plate was incubated for 60 minutes at room temperature, then fluorescence anisotropy was recorded on a CLARIOstar (BMG LabTech) with excitation at 485 nm, emission at 525 nm, and cutoff at 515 nm. Final data were normalized against DMSO control and Bodipy-ATP-γS-free control.

20 SPR for PPIA, including cyclosporine competition

All SPR experiments were performed on a Biacore 8K (Cytiva) instrument at 25°C. For kinetics experiments, the instrument was primed with 10 mM HEPES, pH 7.5, 150 mM NaCl, 0.05% Tween 20, 1 mM DTT, 1% DMSO. A SA Series S sensor chip was docked and pre-conditioned with 3 injections of 50 mM NaOH/1 M NaCl to remove unbound streptavidin from the surface. PPIA protein was diluted to 5 µg/mL in running buffer and immobilized to channels 1 through 8 at 5 µL/min for 50 seconds for a final immobilization level of ~1900 RU. Peptides were diluted to 5 µM in running buffer then serially diluted 2-fold for a total of seven concentrations with one blank (7-point two-fold peptide dilution series with top concentration = 5 µM.). Compounds were injected over the immobilized and reference surfaces at 30 µL/min for 60 seconds then allowed to dissociate for 180 seconds. The surface was regenerated after each cycle with an injection of 1 M sodium chloride. Sensorgrams were double-referenced and fit to a 1:1 steady state affinity model. For ABA competition experiments, PPIA was immobilized similarly to a level of ~850 RU. Compounds were diluted to 10 µM in running buffer and cyclosporine A (CsA) was diluted to 100 nM in running buffer. For each injection, peptides were injected over the surface for 120 seconds at 30 µL/min to achieve equilibrium binding. CsA was then injected for 60 seconds at 30 µL/min in the absence or presence of competing compound over the surface. Surface was regenerated after each cycle with an injection of 1 M sodium chloride. Data was analyzed using Biacore Insight Evaluation software (Cytiva). Sensorgrams were double-referenced and evaluated for competition.

21 PPIA inhibition assay

PPIA inhibition assays were performed at Eurofins Discovery (Ongar, Essex, United Kingdom). Briefly, a 1.5 mL of assay buffer (35 mM HEPES pH 7.8, 50 mM DTT) is pipetted into a 3-mL glass cuvette and cooled to 10°C with stirring. Test compounds are diluted in 100% DMSO then added to the buffer to establish a blank. PPIA is then added at a final concentration of 2 nM and substrate is added to a final concentration of 60 µM. The absorbance at 330 nm is measured for 300 seconds. The resulting data were fit to a first order rate equation and the catalytic rate was calculated. An exponential curve was generated using the catalytic rate versus the inhibitor concentration to obtain a K_i value. CsA is included at a single concentration as a positive control.

22 Production of PDL1

E.coli Protein

For crystallography, human PD-L1 protein (residues 18-134) with a C-terminal 6xHis tag was recombinantly expressed in E. coli BL21 (DE3) CodonPlus RIPL cells (Agilent) from pET-derived expression vectors (Novagen). The cells were induced at OD_{600}=0.6 with 1.0 mM IPTG for 4 hours at 37°C, then harvested and resuspended in 20 mM Tris-HCl pH 8.0, 300 mM NaCl, 10% glycerol, 1 mM PMSF, DNase I. For purification, the pellet was lysed with a tip sonicator, pelleted at 22,000 x g for 50 minutes at 4°C. Protein was located in the inclusion bodies, so the cell pellet was collected and resuspended in 50 mM Tris-HCl pH 8.0, 200 mM NaCl, 10 mM EDTA, 10 mM β-mercaptoethanol (β-ME), 0.5% Triton X-100 and stirred by magnetic stir bar at room temperature for 30 minutes. The suspension was centrifuged at 22,000 x g for 30 minutes

at 4°C. The process was repeated three times. The pellet was resuspended in 50 mM Tris-HCl pH 8.0, 200 mM NaCl, 10 mM EDTA, 10 mM β-ME and stirred by magnetic stir bar at room temperature overnight. The suspension was centrifuged at 22,000 x g for 30 minutes at 4°C and the supernatant was collected. Supernatant was loaded onto a HisTrap (Cytiva) pre-equilibrated with 50 mM Tris-HCl pH 8.0, 200 mM NaCl, 8 M urea, 10 mM β-ME and eluted with 250 mM imidazole. Protein was refolded by diluting into 100 mM Tris-HCl pH 8.0, 1 M L-Arginine, 0.235 mM GSH, 0.25 mM GSSG with incubation overnight at 4°C. Protein was then dialyzed against PBS pH 7.4 for 4 hours at 4°C. Dialysis was repeated three times. Protein was concentrated and injected onto a Superdex™ HiLoad 16/600 75pg SEC column pre-equilibrated with 10 mM Tris-HCl pH 8.0, 20 mM NaCl. Fractions containing pure protein were collected and pooled.

Mammalian Protein
For biochemical assays, human PD-L1 (residues 18-239) with a C-terminal human IgG Fc-Avi™-tag or a C-terminal TEV-10xhis-Avi™ tag were recombinantly co-expressed with BirA in Expi293™ cells from pcDNA-derived plasmid (Thermo Fisher) using the Expifectamine™293 expression system. For Fc-tagged protein, cells were harvested after 5 days of expression and supernatant was collected and passed over a MabSelect sure affinity column (Cytiva) pre-equilibrated with PBS pH 7.4. Protein was eluted with 100 mM sodium citrate, pH 3.0 and neutralized in 1M Tris-base, pH 9.0. Elution was concentrated and injected over a Superdex™ HiLoad 16/600 200pg pre-equilibrated with PBS pH 7.4. Fractions containing pure protein were collected and pooled. For 10xhis-tagged protein, cells were harvested after 5 days of expression and supernatant was collected and passed over a Ni-NTA affinity column (Qiagen) pre-equilibrated with PBS pH 7.4. Protein was eluted with 300 mM imidazole. Elution was concentrated and injected over a Superdex™ HiLoad 16/600 75pg pre-equilibrated with PBS pH 7.4. Fractions containing pure protein were collected and pooled.

23 SPR for PDL1, including PD1 competition

All SPR analysis was performed on a Biacore S200 (Cytiva) in at 25°C. BMS PD-L1 interacting small molecules (BMSpep-57, BMS-1, BMS-1001) were obtained from MedChem Express. A Protein G Series S Sensor Chip (Cytiva) was docked into the instrument primed with PBS pH 7.4 with 0.05% Tween 20 and 1% DMSO. PD-L1-Fc was diluted in running buffer to 50 nM and captured on the surface for 60 seconds at 5 µL/min. Compounds were diluted to 2 µM then serially diluted 3-fold in running buffer (7-point two-fold dilution series with top concentration = 5 µM (FP30790), or 6-point two-fold dilution series with top concentration = 2 µM (others)). Diluted compounds were injected over the surface at 30 µL/min for 180 seconds and allowed to dissociate for 360 seconds. The surface was regenerated every cycle with a 60-second injection of 10 mM glycine-HCl, pH 2.5. The resulting sensorgrams were double-referenced and fit to a 1:1 binding model using Biacore Insight Evaluation software (Cytiva). For ABA competition experiments, PD-L1 was immobilized on a Streptavidin Series S Sensor Chip (Cytiva) to ~250 RU. Compounds were diluted to 10 µM in running buffer and PD-1 was diluted to 400 nM in running buffer. For each injection, compounds were injected over the surface for 120 seconds at 30 µL/min to achieve equilibrium binding. PD-1 was then injected for 60 seconds at 30 µL/min in the absence or presence of competing compound over the surface. The surface was regenerated every cycle with a 60-second injection of 10 mM glycine-HCl, pH 2.5. Data was

analyzed using Biacore Insight Evaluation software (Cytiva). Sensorgrams were double-referenced and evaluated for competition.

24 ELISA for PDL1

PD-1/PD-L1 ELISA competition assays were performed according to manufacturer's instruction (Acro Biosystems). Human PD-L1 was diluted to 2 µg/mL in PBS + 0.05% Tween 20. High-binding 96-well plates (Corning) were coated with PD-L1 at 2 µg/ 100 µL per well. Human PD-1-Avi was diluted to 0.6 µg/mL in ELISA wash buffer (PBS + 0.05% Tween 20 + 0.5% BSA + 0.09% DMSO) and added to the coated wells to form complexes. Test peptides were diluted in ELISA wash buffer to 20 µM then serially diluted 4-fold and added to the PD-L1/PD-1-avi complexes. After incubation with the ligand, the plate was washed, and the bound ligand was detected with the addition of streptavidin-HRP, followed by development with 1-Step™ Ultra TMB-ELISA substrate solution (Thermo Fisher). The HRP reaction was stopped by adding ELISA stop solution (Thermo Fisher). Absorbance at 450 nm was determined on a CLARIOstar plate reader. Samples were blank-subtracted and normalized using in-plate controls. Data were plotted using Prism (Graphpad) and fit to a one-site specific binding model with Hill coefficient.

25 PD-L1 Dimerization Assays, analytical SEC and TR-FRET

Analytical SEC

All analytical SEC methods were performed at Viva Biotech (Shanghai, China) on an Agilent Bio-1260 Infinity II HPLC system. Complexes were prepared by mixing PD-L1 and peptide at a 1:2 protein to peptide ratio. Complexes were injected in separate analyses onto a Superdex® Increase 5/150 200pg column pre-equilibrated with 10mM Tris-HCl pH 8.0, 20mM NaCl containing 1 µM of corresponding peptide (for complexes) or no peptide (for apo-protein). Data was processed using Agilent ChemStation software to determine retention time shifts.

TR-FRET

For TR-FRET dimerization experiments, biotinylated mammalian PD-L1 was diluted to 50 nM, Alexa Fluor™ 488 labeled PD-L1 was diluted to 120 nM and Terbium-labeled streptavidin (Cis-Bio) was diluted to 20 nM in assay buffer (10 mM HEPES pH 7.5, 150 mM NaCl, 0.05% Tween20) in a final volume of 40 µ L per well of a black 384-well plate (Costar). Compounds were serially diluted in 90% DMSO and 80 nL of compound (11-point three-fold peptide dilution series with top concentration = 20 µM) was added to the plate and the samples were incubated for 60 minutes at room temperature. FRET signal was determined using a LanthaScreen™ filter on a PheraStar (BMG Biotech) plate reader (Ex: 337 nm; Em1: 490 nM; Em2: 520 nM). The ratio of Em_{520} to Em_{490} was calculated and plotted against compound concentration. Resulting data was fit to a four-parameter dose-response curve with variable slope.

26 Crystallography

X-Ray Crystallography and Structure Determination. β-catenin/FP01567 complex crystals were obtained in 0.1 M Sodium phosphate monobasic monohydrate, 0.1 M Potassium phosphate monobasic, 0.1 M MES monohydrate pH 6.5, 2.0 M Sodium chloride. β-catenin/FP05874 complex crystals were obtained in 0.05 M $MgCl_2$, 0.1 M MES 6.5 5% (w/v) PEG 4000.

RNF31/FP06649 complex crystals were obtained in 0.03 M Magnesium chloride hexahydrate; 0.03 M Calcium chloride dihydrate, Buffer System 1 pH 6.5 (0.0555M MES, 0.0445 Imidazole), 12.5% MPD, 12.5% PEG 1000, 12.5% PEG 3350. RNF31/FP06652 complex crystals were obtained in 0.02M DL-Glutamic acid monohydrate, 0.02 M DL-Alanine, 0.02 M Glycine, 0.02 M DL-Lysine monohydrochloride, 0.02 M DL-Serine, Buffer System 1 pH 6.5 (0.0555 M MES, 0.0445 M Imidazole), 20% v/v PEG 500 MME, 10 % w/v PEG 20000. RNF31/FP06655 complex crystals were obtained in 10% w/v glycerol, 20% w/v ethanol. CDK2/FP19711 complex crystals were obtained in 0.1 M Tris pH 8.5, 25% (w/v) PEG 2,000 MME. CDK2/FP24322 complex crystals were obtained in 0.1 M Bicine pH 9.2, 2% (v/v) 1,4-Dioxane, 6% (w/v) PEG 20000. PPIA/FP29092 complex crystals were obtained in 0.15 M Potassium bromide, 30% (w/v) Polyethylene glycol monomethyl ether 2,000. PPIA/FP29102 complex crystals were obtained in 0.01 M Nickel (II) chloride hexahydrate, 0.1 M Tris pH 8.5, 20% (w/v) Polyethylene glycol monomethyl ether 2,000. PDL1/FP28135 complex crystals were obtained in 1.1 M Sodium malonate pH 7.0, 0.1 M HEPES pH 7.0, 0.5% (v/v) Jeffamine ED-2001 pH 7.0. PDL1/FP30790 complex crystals were obtained in 0.1 M Sodium acetate pH 4.6, 2.0 M Sodium formate. PPIA/FP29103 complex crystals were obtained in 0.2 M Sodium acetate, 0.1 M Sodium cacodylate pH 6.5, 30% (w/v) PEG 8000. PDL1/FP28132 complex crystals were obtained in 0.1 M MES pH 6.5, 1.6 M Magnesium sulfate. PDL1/FP28136 complex crystals were obtained in 0.1 M TRIS pH 8, 25% (v/v) PEG 350 MME.

Crystals were obtained by either the sitting hanging drop or hanging drop vapor diffusion methods at room temperature. Crystals were cryo-protected followed by flash-freezing in liquid nitrogen. Diffraction datasets were collected at 100K at a variety of sources as described in Dataset S2. Data was processed in XDS & XSCALE (7), AIMLESS (8), and/or STARANISO (9), all part of the autoPROC suite (10). Molecular replacement solutions were obtained using PHASER (11) with previously deposited high resolution PDB structures as search models. Complete models were built through iterative cycles of manual model building in COOT (12) and structure refinement was carried out using either REFMAC (13) or PHENIX (14). All the structure model figures in the paper were prepared using PyMOL (The PyMOL Molecular Graphics System, Version 2.4, Schrödinger, LLC.). The atomic coordinates and structure factors have been deposited in the Protein Data Bank, www.pdb.org.

Supporting Information Figures

A *Characterization of Stapled and Unstapled FP01567*

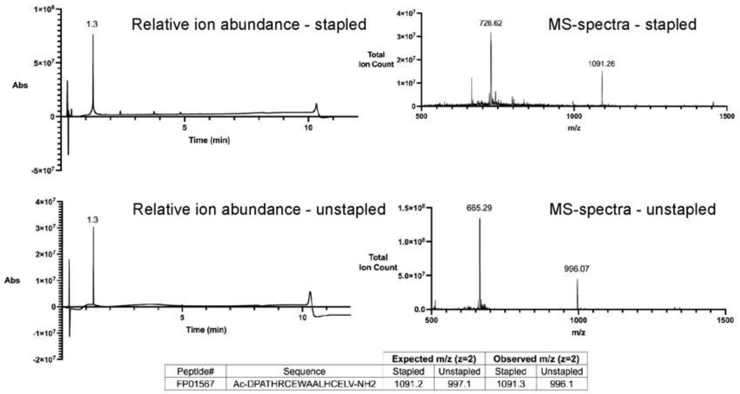

		Expected m/z (z=2)		Observed m/z (z=2)	
Peptide#	Sequence	Stapled	Unstapled	Stapled	Unstapled
FP01567	Ac-DPATHRCEWAALHCELV-NH2	1091.2	997.1	1091.3	996.1

B *Characterization of Stapled and Unstapled FP06641*

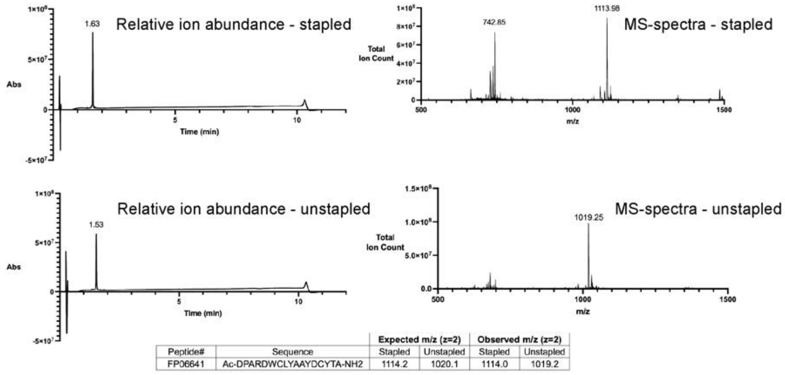

		Expected m/z (z=2)		Observed m/z (z=2)	
Peptide#	Sequence	Stapled	Unstapled	Stapled	Unstapled
FP06641	Ac-DPARDWCLYAAYDCYTA-NH2	1114.2	1020.1	1114.0	1019.2

C *Characterization of Stapled and Unstapled FP06635*

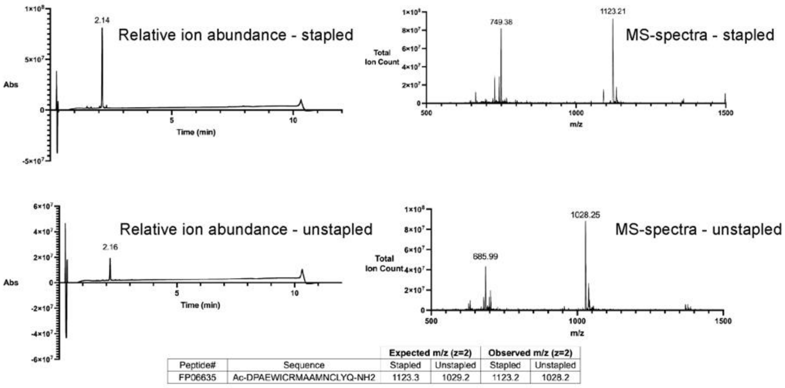

		Expected m/z (z=2)		Observed m/z (z=2)	
Peptide#	Sequence	Stapled	Unstapled	Stapled	Unstapled
FP06635	Ac-DPAEWICRMAAMNCLYQ-NH2	1123.3	1029.2	1123.2	1028.2

Figure S1. Characterization of β-catenin- and RNF31-binding Helicons.
Analytical HPLC (left traces) and mass spectrometry (right spectra and tables) of stapled and unstapled (prior to stapling reaction) Helicons that bind to β-catenin (A), and the PUB domain of RNF31 (B-C).

A *Mass Spectrum of Axin Clone Crosslinked on Phage*

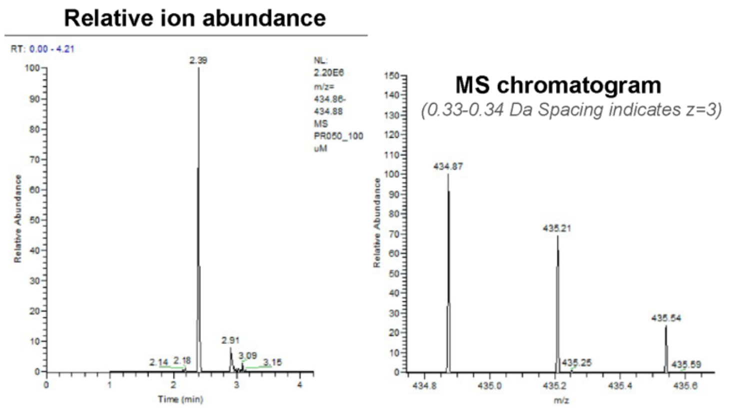

Cleaved sequence	Expected crosslinked mass	Expected M+3/3	Observed M+3/3
ACILDAHICR	1301.601 Da	434.874 Da	434.87 Da

B *Sequencing of Phage Colonies*

Observed Sequence	Match Library Design?
DPAWHQCYVAAMLCDNE	Yes
DPASQHCLYAAVRCDER	Yes
DPANSECITAAYICTLE	Yes
DPAWDVFCYNAADYCYSD	No - 1 residue insertion
DPAVELCQDAAQICYMS	Yes
DPAVRECEMAARWCFEW	Yes
DPAANDCIYAAMLCYTF	Yes
DPAEEVCNLAAHECRWF	Yes
DPASYMCVQAASHCLSM	Yes
DPATQMCLNAAQSCMHI	Yes
DPAAQHCETAAYHCIFY	Yes
DPAMRHCLHAAYLCDQV	Yes

Figure S2. Characterization of a crosslinked peptide displayed on phage. (A) Analytical HPLC and mass spectrometry of a crosslinked phage clone confirms on-phage cysteine stapling. The retention times in minutes are indicated above each of the relevant peptide peaks. (B) Sequencing of 12 phage library members indicates that 11 of 12 sequences match the expected library design, with one sequence containing an additional amino acid insertion.

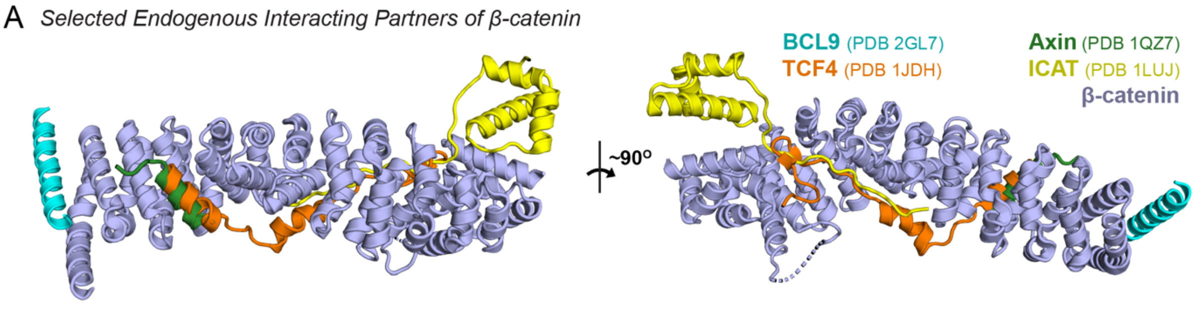

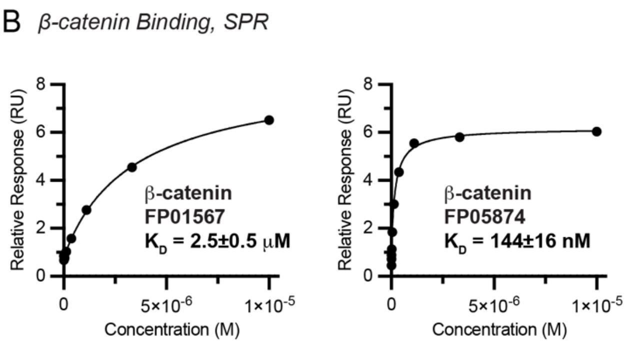

C Alanine Scanning of FP01567

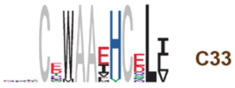

Peptide ID	Sequence	K_D (μM, SPR)
**FP01567**	DPATHRCEWAALHCELV	2.5±0.50
FP49250	DPAAHRCEWAALHCELV	1.4±0.55
FP49251	DPATARCEWAALHCELV	0.4±0.05
FP49252	DPATHACEWAALHCELV	0.3±0.10
FP49253	DPATHRCAWAALHCELV	2.7±1.53
FP49254	DPATHRCEAAALHCELV	>20
FP49255	DPATHRCEWAAAHCELV	>20
FP49256	DPATHRCEWAALACELV	*n.d.*
FP49257	DPATHRCEWAALHCALV	1.0*
FP49258	DPATHRCEWAALHCEAV	>20
FP49259	DPATHRCEWAALHCELA	>20

*no replicate data

Figure S3. Binding properties of β-catenin
(A) Two opposite faces of the β-catenin ARM domain structure (lilac, PDB 1QZ7) are shown overlaid with several of its binding partners, as determined by x-ray crystallography for BCL9 (15), TCF4 (16), Axin (17), and ICAT (18). (B) Representative surface plasmon resonance (SPR) analysis of the interaction of Helicons with immobilized β-catenin (K_D values and SD based on triplicates). The responses observed in sensorgrams for each concentration of Helicon were plotted to determine the binding constant (K_D). (C) SPR analysis of cluster C33 Helicon FP01567 and an alanine-scanning series across the non-crosslinked residues of the peptide demonstrates the importance of the conserved logo residues for binding (n=2 or 3; data are presented as mean ± SD). *n.d.*, no data.

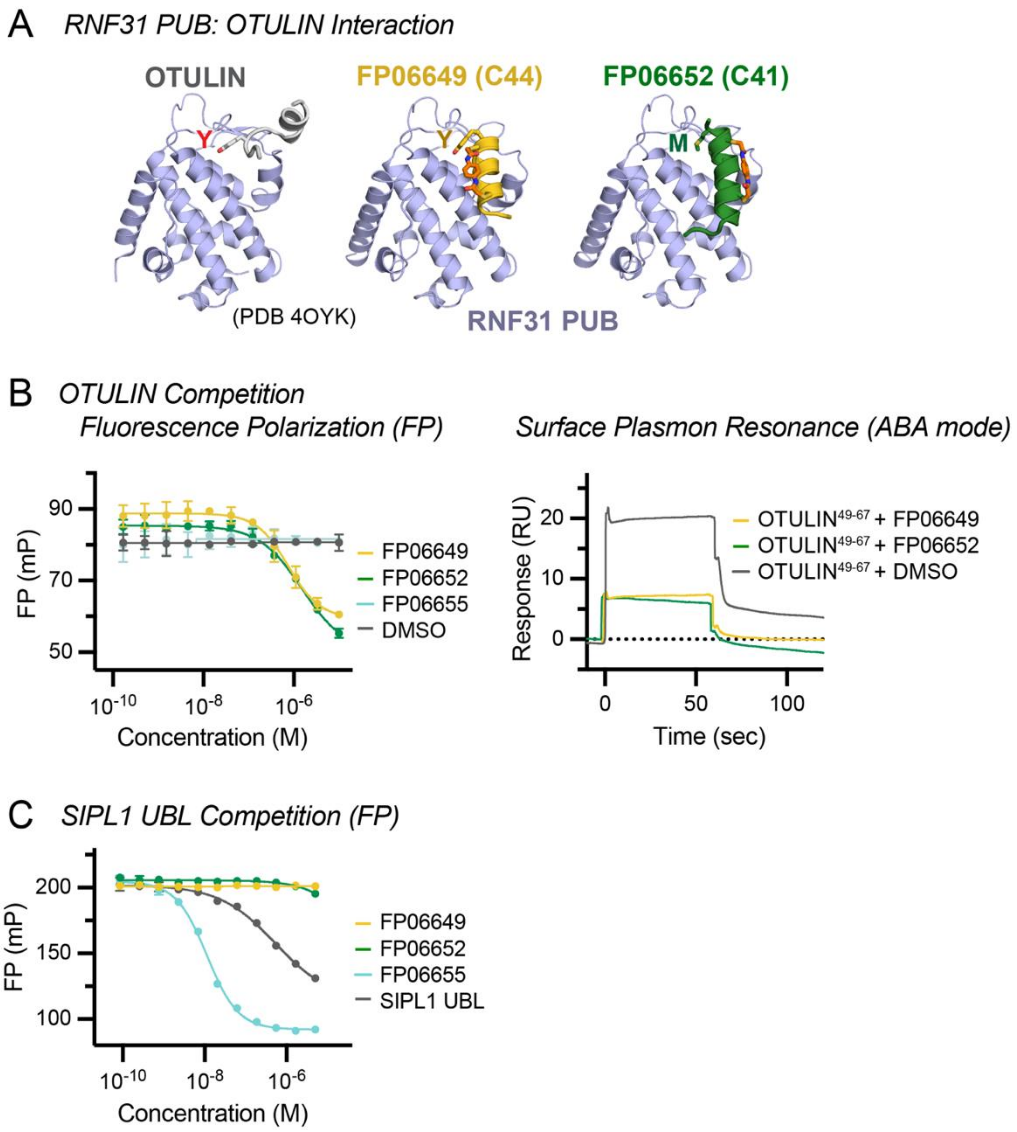

Figure S4. Structural and functional analyses of RNF31 PUB- and UBA-binding Helicons. (A) Cluster C44 Helicon FP06649 and Cluster C41 Helicon FP06652 share an RNF31 UBL-binding site, but bind with different orientations compared to Otulin, as shown by x-ray crystallography (19). (B) Helicons FP06649 and FP06652 compete with fluorescently labeled Otulin peptide for binding to RNF31-PUB as monitored by fluorescence polarization, while the RNF31 UBA-binding helicon, FP06655 does not compete (n=2; data are presented as mean ± SD), and by Surface Plasmon Resonance (SPR, Biacore). RNF31 UBA-binding Helicon FP06655 from Cluster C36 (Fig. 4D, E) does not compete for binding to the Otulin site on RNF31 PUB. (C) Helicon FP06655 and unlabeled Sharpin/SIPL1 UBL domain compete with fluorescently labeled 5FAM-FP06655 for binding to RNF31-UBA, while the RNF31-PUB binding peptides (FP06649 and FP06652) do not compete (n=3; data are presented as mean ± SD).

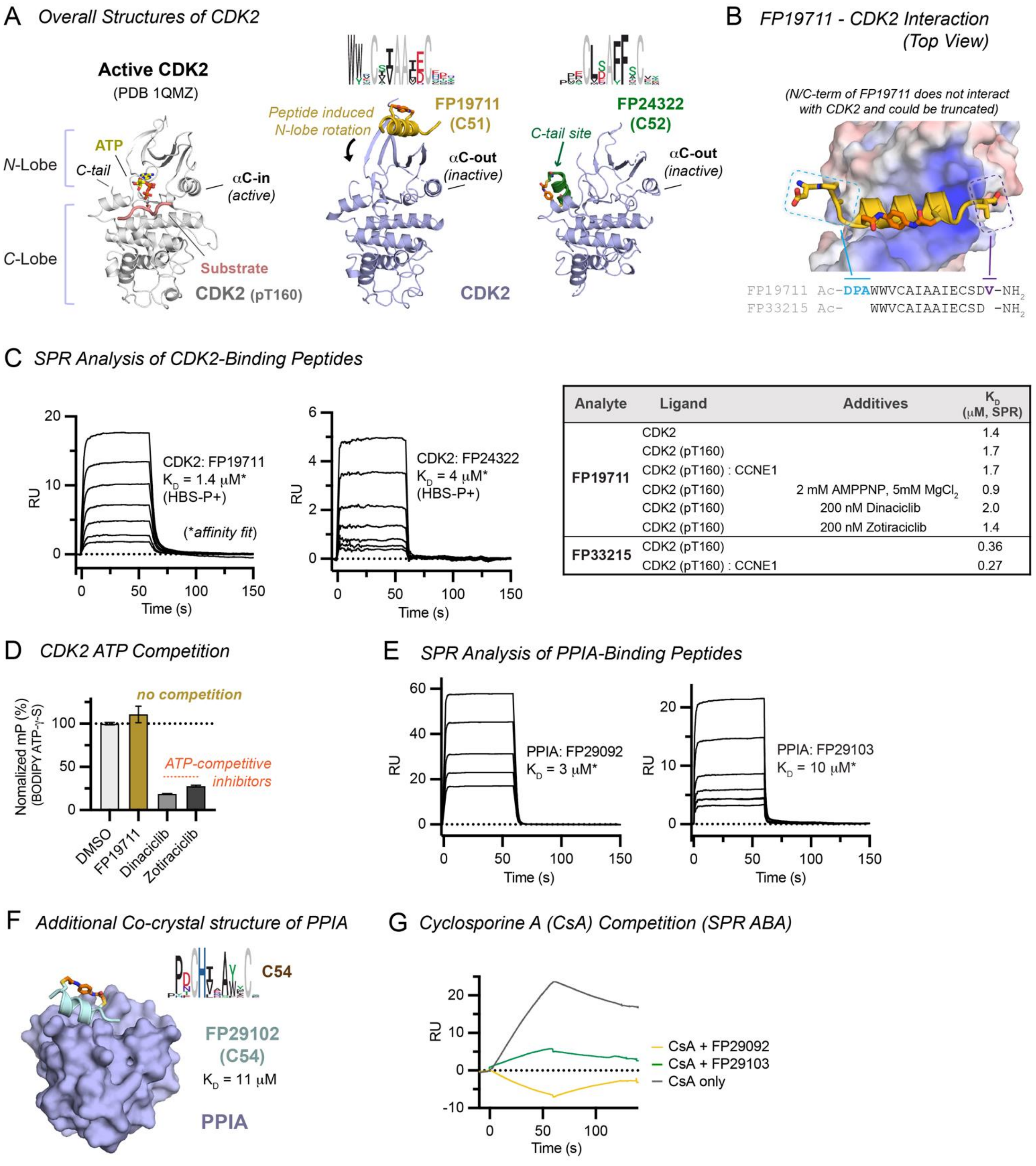

Analyte	Ligand	Additives	K_D (μM, SPR)
FP19711	CDK2		1.4
	CDK2 (pT160)		1.7
	CDK2 (pT160) : CCNE1		1.7
	CDK2 (pT160)	2 mM AMPPNP, 5mM $MgCl_2$	0.9
	CDK2 (pT160)	200 nM Dinaciclib	2.0
	CDK2 (pT160)	200 nM Zotiraciclib	1.4
FP33215	CDK2 (pT160)		0.36
	CDK2 (pT160) : CCNE1		0.27

Figure S5. Structural and functional analyses of CDK2- and PPIA-binding Helicons. (A) Cluster C51 peptide FP19711 and Cluster C52 peptide FP24322 bind distinct sites from the orthosteric CDK2 ATP site (20), and FP19711 induces a rotation of the N-terminal lobe. (B) The FP19711-CDK2 co-structure indicates that the N- and C-terminal-most residues of the peptide do not

interact with CDK2 (seen also in Fig. S7). Truncation of FP19711 to FP33215 improves its CDK2-binding affinity to ~300 nM. (C) By SPR (Biacore), FP19711 and FP24322 bind CDK2. FP19711 and its truncated version, FP33215, also bind the active CDK2 (with T160 phosphorylated) and active CDK2: Cyclin E1 (CCNE1) complex. In addition, FP19711 retains its CDK2-binding affinity in the presence of ATP-analog (AMPPNP) or ATP-competitive inhibitors (Dinaciclib and Zotiraciclib), suggesting it is not ATP-competitive. (D) Compared with ATP-competitive CDK2 inhibitors, FP19711 does not compete with fluorescently labeled ATP analog (BODIPY-ATP-γS) for binding CDK2 as assessed by fluorescence polarization. This is consistent with its binding to an allosteric site on CDK2. (E) By SPR (Biacore), Cluster C53 peptide FP29092 and Cluster C54 peptide FP29103 bind to PPIA. (F) Co-crystal structure of Cluster C54 peptide FP29102 Helicon with PPIA, like that of FP29103 (Fig. 5D), shows that it binds a site similar to that of Cyclosporine A and peptide substrates. (G) Helicons FP29092 and FP29103 compete with Cyclosporine A for binding to PPIA as monitored by Surface Plasmon Resonance (SPR, Biacore).

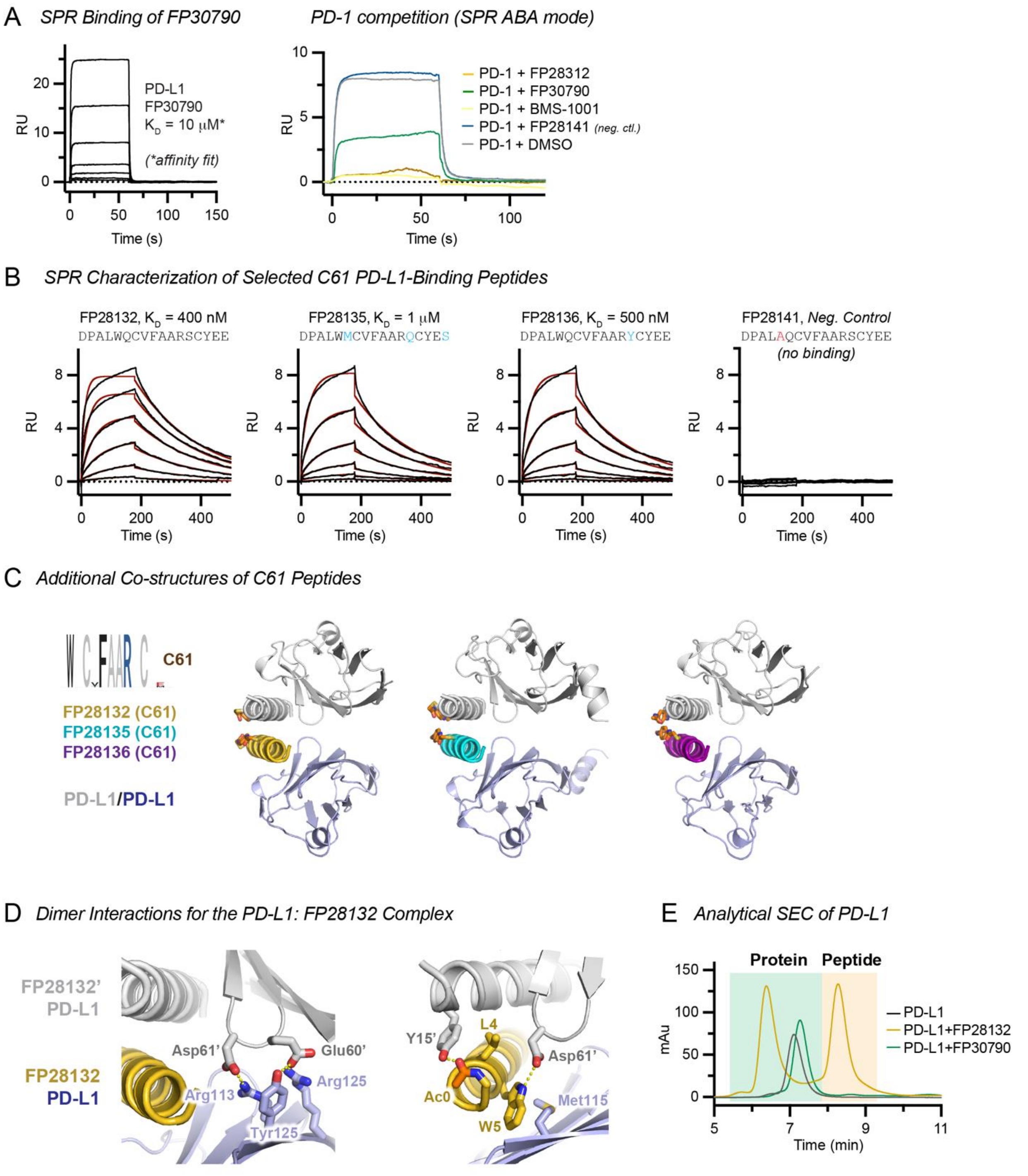

Figure S6. Characterization of PD-L1-binding Helicons (A) SPR binding and competition assays show that Cluster C62 peptide FP30790 binds to PD-L1 (left) and while it competes for binding to PD-L1 with PD-L1 receptor PD-1, Cluster C61 Helicon FP28312 and the small-molecule inhibitor of the PD-1/PD-L1 interaction, BMS-1001 compete more potently (right). FP28141 is a point mutant of FP28132 with an alanine residue replacing a conserved tryptophan from the

phage cluster logo, that does not bind PD-L1 (see S6B). (B) Surface plasmon resonance (SPR, Biacore) shows binding of FP28132 and additional Cluster C61 Helicons FP28135 and FP28136 to PD-L1, while FP28141 does not bind. (C) As with FP28132 (here and in Fig 6B), the co-crystal structure of FP28135 and FP28136 with PD-L1 shows a symmetric dimer of two Helicon/PD-L1 complexes. (D) Close examination of the co-structure reveals an extensive series of contacts between both the two FP28132 protomers and the two PD-L1 protomers. (E) Analytical SEC suggests that the apo-PD-L1 and FP30790-PD-L1 are monomeric, while FP28132-PD-L1 complex is a dimer in solution.

CTNNB1: FP01567

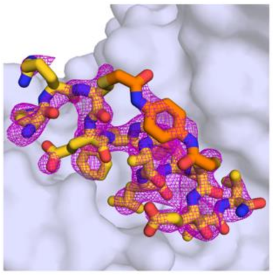

CTNNB1: FP05874

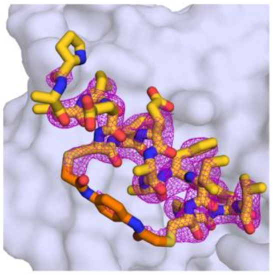

RNF31 PUB: FP06649

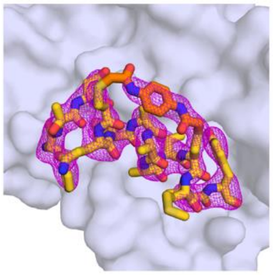

RNF31 PUB: FP06652

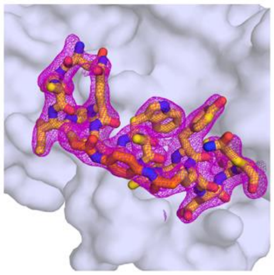

RNF31 UBA: FP06655

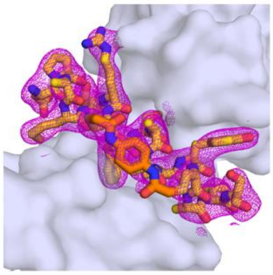

CDK2: FP19711

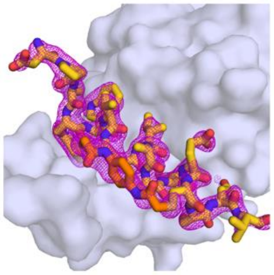

CDK2: FP24322

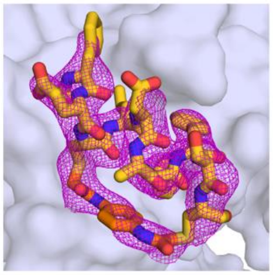

PPIA: FP29092

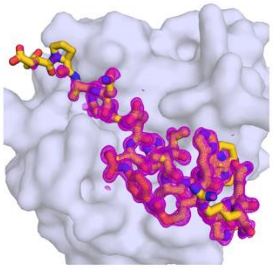

PPIA: FP29102

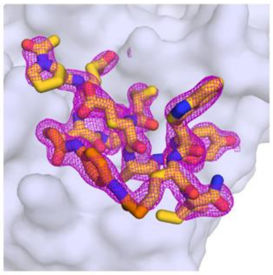

PPIA: FP29103

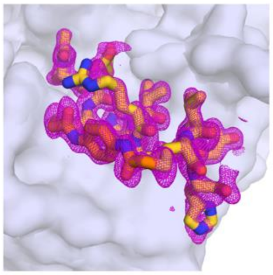

PD-L1: FP28132

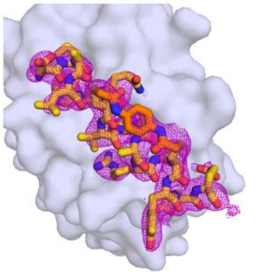

PD-L1: FP28135

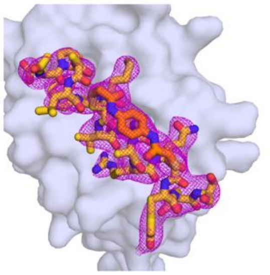

PD-L1: FP28136

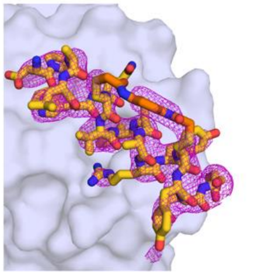

PD-L1: FP30790

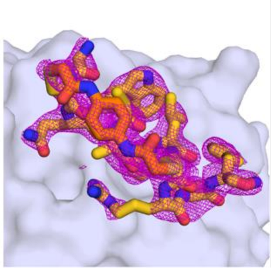

Figure S7. Composite omit maps (contour level: 1.0 σ) of Helicons bound to their target sites on the six protein domains studied in this work.

Supporting Information Tables

Table S1. Quantitation of peptide helicity before and after stapling

Peptide ID	Target	Helicity unstapled	Helicity stapled
FP01848	β-catenin (C31)	39%	75%
FP49193	β-catenin (C32)	30%	50%
FP01567	β-catenin (C33)	27%	51%
FP01822	β-catenin (C33)	34%	72%
FP49200	β-catenin (C34)	22%	66%
FP06641	RNF31 PUB (C43)	16%	41%
FP06635	RNF31 PUB (C49)	23%	52%

Table S2. Assessment of the cell-associated fraction for a panel of β-catenin-binding Helicons

FOG ID	Cluster	Parent peptide sequence	Helicon in cells[a] ± SD (%)	β-catenin Binding IC_{50}[b] (TCF probe, uM)	CellTiter-Glo fold change[c] ± SD
**FP47839**	(Assay Positive Control)	5FAM-SAH-p53-8[d] (21)	2.1 ± 0.1		1.3 ± 0.01
**FP01413**	(Assay Positive Control)	ATSP-7041[d] (22)	3.7 ± 0.4		1.2 ± 0.07
**FP47831**	(Assay Negative Control)	5FAM-PKCI[d] (23)	0.0 ± 0.0		1.1 ± 0.02
**FP47842**	(Assay Negative Control)	5FAM-D-Myc peptide[d]	0.0 ± 0.0		1.2 ± 0.01
**FP47843**	(Assay Negative Control)	5FAM-D-HA peptide[d]	0.0 ± 0.0		1.2 ± 0.02
**FP07855**	C35 (CTNNB1)	DPAVMECYEAAFICHYV	16.4 ± 0.6	4.0 ± 0.1	1.2 ± 0.10
**FP07856**	C35 (CTNNB1)	DPAVMECYEAAFICHYV	15.5 ± 0.9	6.6 ± 0.9	1.3 ± 0.12
**FP07859**	C35 (CTNNB1)	DPAILQCYEAAFSCHYQ	0.1 ± 0.0	>10	1.2 ± 0.02
**FP07860**	C35 (CTNNB1)	DPAILQCYEAAFSCHYQ	0.3 ± 0.0	>10	1.2 ± 0.03
**FP07863**	C35 (CTNNB1)	DPAVMECYEAAFICHYV	23.5 ± 6.5	8.7 ± 1.6	1.2 ± 0.02
**FP07864**	C35 (CTNNB1)	DPAVMECYEAAFICHYV	18.5 ± 11.4	7.6 ± 1.1	1.1 ± 0.05
**FP07867**	C35 (CTNNB1)	DPAVMECYEAAFICHYV	8.6 ± 0.4	>10	1.2 ± 0.03
**FP07868**	C35 (CTNNB1)	DPAVMECYEAAFICHYV	10.3 ± 1.4	>10	1.2 ± 0.10
**FP07871**	C35 (CTNNB1)	DPAVMECYEAAFICHYV	7.0 ± 0.3	>10	1.2 ± 0.03
**FP07872**	C35 (CTNNB1)	DPAVMECYEAAFICHYV	11.7 ± 1.6	>10	1.1 ± 0.02

**FP08011**	C35 (CTNNB1)	DPAIIHCYEAAFFCQYI	6.5 ± 0.5	>10	1.2 ± 0.01
**FP08012**	C35 (CTNNB1)	DPAIIHCYEAAFFCQYI	5.4 ± 0.9	>10	1.1 ± 0.04
**FP08017**	C35 (CTNNB1)	DPAILECYEAAFECQYM	0.6 ± 0.1	>10	1.2 ± 0.01
**FP08018**	C35 (CTNNB1)	DPAILECYEAAFECQYM	1.0 ± 0.1	>10	1.1 ± 0.05
**FP08023**	C35 (CTNNB1)	DPAIMACYQAAFWCQYN	3.0 ± 0.3	>10	1.2 ± 0.03
**FP08024**	C35 (CTNNB1)	DPAIMACYQAAFWCQYN	6.5 ± 0.5	>10	1.2 ± 0.00
**FP08029**	C35 (CTNNB1)	DPAILTCYEAAFTCQYQ	0.2 ± 0.0	8.0 ± 1.1	1.1 ± 0.03
**FP08030**	C35 (CTNNB1)	DPAILTCYEAAFTCQYQ	0.3 ± 0.0	>10	1.2 ± 0.02

a. The percentage of Helicons in cells after treatment and wash (see Materials and Methods).
n=2; data are presented as mean ± SD.
b. *In vitro* assay for competition with β-catenin-binding TCF peptide, as in Figure 3.
c. Measure of ATP levels, reflective of cell health (see Materials and Methods).
d. Full sequences of control peptides can be found in Table S3.

Table S3. List of all peptides and targets described in this work with expected and observed masses.

Target	FOG ID	Sequence	Expected Mass	Observed Mass	Staple type
**CTNNB1**	FP01567	Ac-DPATHRCEWAALHCELV-NH2	2180.4	2180.2	Cys-stapled
**CTNNB1**	FP49332	Ac-DPATHRCEWAALHCELV-NH2	1992.2	1989.2	unstapled
**CTNNB1**	FP49250	Ac-DPAAHRCEWAALHCELV-NH2	2150.4	2149.0	Cys-stapled
**CTNNB1**	FP49251	Ac-DPATARCEWAALHCELV-NH2	2114.4	2112.8	Cys-stapled
**CTNNB1**	FP49252	Ac-DPATHACEWAALHCELV-NH2	2095.3	2094.0	Cys-stapled
**CTNNB1**	FP49253	Ac-DPATHRCAWAALHCELV-NH2	2122.4	2120.8	Cys-stapled
**CTNNB1**	FP49254	Ac-DPATHRCEAAALHCELV-NH2	2065.3	2063.8	Cys-stapled
**CTNNB1**	FP49255	Ac-DPATHRCEWAAAHCELV-NH2	2138.3	2136.8	Cys-stapled
**CTNNB1**	FP49257	Ac-DPATHRCEWAALHCALV-NH2	2122.4	2120.8	Cys-stapled
**CTNNB1**	FP49258	Ac-DPATHRCEWAALHCEAV-NH2	2138.3	2136.8	Cys-stapled
**CTNNB1**	FP49259	Ac-DPATHRCEWAALHCELA-NH2	2152.4	2150.8	Cys-stapled
**CTNNB1**	FP01822	Ac-DPASELCEWAAIHCDLV-NH2	2101.3	2100.2	Cys-stapled
**CTNNB1**	FP49333	Ac-DPASELCEWAAIHCDLV-NH2	1913.1	1912.1	unstapled
**CTNNB1**	FP01838	Ac-DPAILECHIAAWNCYEI-NH2	2190.5	2189.0	Cys-stapled
**CTNNB1**	FP49194	Ac-DPATLDCHIAAWDCWDE-NH2	2190.3	2188.8	Cys-stapled
**CTNNB1**	FP49193	Ac-DPAILACHLAAMDCSDW-NH2	2061.3	2060.4	Cys-stapled
**CTNNB1**	FP49432	Ac-DPAILACHLAAMDCSDW-NH2	1873.1	1871.2	unstapled
**CTNNB1**	FP49196	Ac-DPANANCILAAHECRIW-NH2	2126.4	2124.8	Cys-stapled
**CTNNB1**	FP49197	Ac-DPAEVECMLAAHVCRAF-NH2	2091.4	2089.8	Cys-stapled
**CTNNB1**	FP01848	Ac-DPAQDDCILAAHVCALW-NH2	2070.3	2069.1	Cys-stapled
**CTNNB1**	FP49433	Ac-DPAQDDCILAAHVCALW-NH2	1882.1	1881.1	unstapled
**CTNNB1**	FP49199	Ac-DPADWECEHAALLCHYW-NH2	2288.5	2286.9	Cys-stapled
**CTNNB1**	FP49200	Ac-DPALWQCEHAALLCDVH-NH2	2150.4	2149.2	Cys-stapled
**CTNNB1**	FP49431	Ac-DPALWQCEHAALLCDVH-NH2	1962.2	1959.5	
**CTNNB1**	FP05863	Ac-DPAIIHCYEAAFFCQYI-NH2	2233.5	2233.4	Cys-stapled
**CTNNB1**	FP05874	Ac-DPAVMECYEAAFICHYV-NH2	2190.5	2189.3	Cys-stapled
**CTNNB1**	FP04872	5FAM-bAla-DDLGANDELISFKDEGEQEEKSSENSSAERDLADVKSSLVNESE-NH2	5245.3	5242.2	Cys-stapled
**CTNNB1**	FP00013 (fStAx-33 (5))	FITC-PEG1-PQ-S5-ILD-S5-HVRRVWR	2358.8	2357.8	S5-S5 stapled
**RNF31 (PUB)**	FP06635	Ac-DPAEWICRMAAMNCLYQ-NH2	2244.6	2244.4	Cys-stapled
**RNF31 (PUB)**	FP49434	Ac-DPAEWICRMAAMNCLYQ-NH2	2056.4	2054.2	unstapled
**RNF31 (PUB)**	FP06641	Ac-DPARDWCLYAAYDCYTA-NH2	2226.4	2226.3	Cys-stapled
**RNF31 (PUB)**	FP49435	Ac-DPARDWCLYAAYDCYTA-NH2	2038.2	2035.9	unstapled
**RNF31 (PUB)**	FP06649	Ac-DPAFTDCQLAAAVCMTY-NH2	2049.3	2049.5	Cys-stapled
**RNF31 (PUB)**	FP06652	Ac-DPAIVQCAWAALYCDMQ-NH2	2127.4	2127.3	Cys-stapled

**RNF31 (PUB)**	FP16923	FITC-NHHex-AEHEEDMYRAADEIEKEKE-NH2	2824.0	2822.0	Cys-stapled
**RNF31 (UBA)**	FP06655	Ac-DPAMQRCFSAAVYCAIS-NH2	2062.3	2062.3	Cys-stapled
**RNF31 (UBA)**	FP12122	5FAM-bAla-DPAMQRCFSAAVYCAIS-NH2	2449.7	2449.8	Cys-stapled
**CDK2**	FP24322	Ac-FECLDAFFSC-NH2	1410.6	1409.8	Cys-stapled
**CDK2**	FP19711	Ac-DPAWWVCAIAAIECSDV-NH2	2078.3	2077.4	Cys-stapled
**CDK2**	FP33215	Ac-WWVCAIAAIECSD-NH2	1695.9	1695.0	Cys-stapled
**PPIA**	FP29103	Ac-PDCHIRAYVCH-NH2	1542.7	1542.1	Cys-stapled
**PPIA**	FP29092	Ac-DPANQDCHVAAWHCWQR-NH2	2266.4	2265.8	Cys-stapled
**PPIA**	FP29102	Ac-PECHIEAYWCI-NH2	1592.8	1591.8	Cys-stapled
**PD-L1**	FP30790	Ac-DPAAADCQWAAFLCRVY-NH2	2129.4	2128.0	Cys-stapled
**PD-L1**	FP28132	Ac-DPALWQCVFAARSCYEE-NH2	2217.4	2217.0	Cys-stapled
**PD-L1**	FP28141	Ac-DPALAQCVFAARSCYEE-NH2	2102.3	2102.5	Cys-stapled
**PD-L1**	FP28135	Ac-DPALWMCVFAARQCYES-NH2	2219.5	2218.9	Cys-stapled
**PD-L1**	FP28136	Ac-DPALWQCVFAARYCYEE-NH2	2293.5	2293.2	Cys-stapled
**PRKCI (5FAM-PKC iota/zeta-blocking peptide)**	FP47831	5FAM-QRFARKGALRQKNV-NH2	2028.0	2029.2	unstapled
**NA (F5AM-D-Myc peptide)**	FP47842	5FAM-dGlu-dGln-dLys-dLeu-dIle-dSer-dGlu-dGlu-dAsp-dLeu-NH2	1559.7	1561.1	unstapled
**NA (5FAM-D-HA peptide)**	FP47843	5FAM-dTyr-dPro-dTyr-dAsp-dVal-dPro-dAsp-dTyr-dAla-NH2	1458.5	1459.8	unstapled
**MDM2 (5FAM-SAH-p53-8)**	FP47839	Ac-[5FAM]-Lys-Gln-Ser-Gln-Gln-Thr-Phe-R8-Asn-Leu-Trp-Arg-Leu-Leu-S5-Gln-Asn-NH2	2594.3	2596.4	R8-S5 stapled (24)
**MDM2 (ATSP-7041)**	FP01413	Ac-Leu-Thr-Phe-R8-Glu-Tyr-Trp-Ala-Gln-Cba-S5-Ser-Ala-Ala-NH2	1743.9	1745.0	R8-S5 stapled
**CTNNB1**	FP07855	Ac-Val-nLeu-Glu-R8-Tyr-Glu-Ala-Ala-Phe-Ile-S5-His-Tyr-Val-NH2	1786.0	1785.7	R8-S5 stapled
**CTNNB1**	FP07856	Ac-Val-nLeu-Glu-R8-Tyr-Glu-Ala-Ala-Phe-Ile-S5-His-Tyr-Val-NH2	1786.0	1785.8	R8-S5 stapled
**CTNNB1**	FP07859	Ac-Ile-Leu-Gln-R8-Tyr-Glu-Ala-Ala-Phe-Ser-S5-His-Tyr-Gln-NH2	1801.9	1801.6	R8-S5 stapled
**CTNNB1**	FP07860	Ac-Ile-Leu-Gln-R8-Tyr-Glu-Ala-Ala-Phe-Ser-S5-His-Tyr-Gln-NH2	1801.9	1801.7	R8-S5 stapled

**CTNNB1**	FP07863	Ac-Val-nLeu-Gln-R8-Tyr-Glu-Ala-Ala-Phe-Ile-S5-His-Tyr-Val-NH2	1785.0	1784.8	R8-S5 stapled
**CTNNB1**	FP07864	Ac-Val-nLeu-Gln-R8-Tyr-Glu-Ala-Ala-Phe-Ile-S5-His-Tyr-Val-NH2	1785.0	1784.8	R8-S5 stapled
**CTNNB1**	FP07867	Ac-Val-nLeu-Glu-R8-Tyr-Gln-Ala-Ala-Phe-Ile-S5-His-Tyr-Val-NH2	1785.0	1784.8	R8-S5 stapled
**CTNNB1**	FP07868	Ac-Val-nLeu-Glu-R8-Tyr-Gln-Ala-Ala-Phe-Ile-S5-His-Tyr-Val-NH2	1785.0	1784.8	R8-S5 stapled
**CTNNB1**	FP07871	Ac-Val-nLeu-Gln-R8-Tyr-Gln-Ala-Ala-Phe-Ile-S5-His-Tyr-Val-NH2	1784.0	1783.8	R8-S5 stapled
**CTNNB1**	FP07872	Ac-Val-nLeu-Gln-R8-Tyr-Gln-Ala-Ala-Phe-Ile-S5-His-Tyr-Val-NH2	1784.0	1783.8	R8-S5 stapled
**CTNNB1**	FP08011	Ac-Ile-Ile-His-R8-Tyr-Glu-Ala-Ala-Phe-Phe-S5-Gln-Tyr-Ile-NH2	1847.0	1847.2	R8-S5 stapled
**CTNNB1**	FP08012	Ac-Ile-Ile-His-R8-Tyr-Glu-Ala-Ala-Phe-Phe-S5-Gln-Tyr-Ile-NH2	1847.0	1847.4	R8-S5 stapled
**CTNNB1**	FP08017	Ac-Ile-Leu-Glu-R8-Tyr-Glu-Ala-Ala-Phe-Glu-S5-Gln-Tyr-nLeu-NH2	1821.0	1821.1	R8-S5 stapled
**CTNNB1**	FP08018	Ac-Ile-Leu-Glu-R8-Tyr-Glu-Ala-Ala-Phe-Glu-S5-Gln-Tyr-nLeu-NH2	1821.0	1821.1	R8-S5 stapled
**CTNNB1**	FP08023	Ac-Ile-nLeu-Ala-R8-Tyr-Gln-Ala-Ala-Phe-Trp-S5-Gln-Tyr-Asn-NH2	1820.0	1820.1	R8-S5 stapled
**CTNNB1**	FP08024	Ac-Ile-nLeu-Ala-R8-Tyr-Gln-Ala-Ala-Phe-Trp-S5-Gln-Tyr-Asn-NH2	1820.0	1820.1	R8-S5 stapled
**CTNNB1**	FP08029	Ac-Ile-Leu-Thr-R8-Tyr-Glu-Ala-Ala-Phe-Thr-S5-Gln-Tyr-Gln-NH2	1779.9	1780.1	R8-S5 stapled
**CTNNB1**	FP08030	Ac-Ile-Leu-Thr-R8-Tyr-Glu-Ala-Ala-Phe-Thr-S5-Gln-Tyr-Gln-NH2	1779.9	1780.1	R8-S5 stapled

Dataset S1. List of the top 20 phage screening hits for each cluster. Phage binding data are listed for C31-35 (β-catenin), C41-49 (RNF31), C51-52 (CDK2), C53-54 (PPIA), and C61-62 (PD-L1). Some clusters contained fewer than 20 hits.

Dataset S2. Data collection and refinement statistics for all structures reported in this work. This table also includes beamline acknowledgements.

Supporting Information References

1. N. J. Greenfield, Using circular dichroism spectra to estimate protein secondary structure. *Nat Protoc* 1, 2876–2890 (2006).

2. N. E. Shepherd, H. N. Hoang, G. Abbenante, D. P. Fairlie, Single Turn Peptide Alpha Helices with Exceptional Stability in Water. *J Am Chem Soc* 127, 2974–2983 (2005).

3. D. Wang, K. Chen, J. L. Kulp, P. S. Arora, Evaluation of Biologically Relevant Short α-Helices Stabilized by a Main-Chain Hydrogen-Bond Surrogate. *J Am Chem Soc* 128, 9248–9256 (2006).

4. P. Imming, "Medicinal Chemistry: Definitions and Objectives, Drug Activity Phases, Drug Classification Systems" in *The Practice of Medicinal Chemistry, Fourth Edition*, C. Wermuth, D. Aldous, P. Raboisson, D. Rognan, Eds. (2015).

5. T. N. Grossmann, *et al.*, Inhibition of oncogenic Wnt signaling through direct targeting of β-catenin. *Proc National Acad Sci* 109, 17942–17947 (2012).

6. J. Yin, *et al.*, Genetically encoded short peptide tag for versatile protein labeling by Sfp phosphopantetheinyl transferase. *Proc National Acad Sci* 102, 15815–15820 (2005).

7. W. Kabsch, Integration, scaling, space-group assignment and post-refinement. *Acta Crystallogr Sect D Biological Crystallogr* 66, 133–144 (2010).

8. P. R. Evans, G. N. Murshudov, How good are my data and what is the resolution? *Acta Crystallogr Sect D Biological Crystallogr* 69, 1204–1214 (2013).

9. I. J. Tickle, *et al.*, Staraniso (Global Phasing, Ltd., Cambridge, United Kingdom) (2018).

10. C. Vonrhein, *et al.*, Data processing and analysis with the autoPROC toolbox. *Acta Crystallogr Sect D Biological Crystallogr* 67, 293–302 (2011).

11. A. J. McCoy, *et al.*, Phaser crystallographic software. *J Appl Crystallogr* 40, 658–674 (2007).

12. P. Emsley, B. Lohkamp, W. G. Scott, K. Cowtan, Features and development of Coot. *Acta Crystallogr Sect D Biological Crystallogr* 66, 486–501 (2010).

13. G. N. Murshudov, *et al.*, REFMAC5 for the refinement of macromolecular crystal structures. *Acta Crystallogr Sect D Biological Crystallogr* 67, 355–367 (2011).

14. D. Liebschner, *et al.*, Macromolecular structure determination using X-rays, neutrons and electrons: recent developments in Phenix. *Acta Crystallogr Sect D* 75, 861–877 (2019).

15. J. Sampietro, *et al.*, Crystal Structure of a β-Catenin/BCL9/Tcf4 Complex. *Mol Cell* 24, 293–300 (2006).

16. T. A. Graham, D. M. Ferkey, F. Mao, D. Kimelman, W. Xu, Tcf4 can specifically recognize β-catenin using alternative conformations. *Nat Struct Biol* 8, 1048–1052 (2001).

17. Y. Xing, W. K. Clements, D. Kimelman, W. Xu, Crystal structure of a β-catenin/Axin complex suggests a mechanism for the β-catenin destruction complex. *Gene Dev* 17, 2753–2764 (2003).

18. T. A. Graham, W. K. Clements, D. Kimelman, W. Xu, The Crystal Structure of the β-Catenin/ICAT Complex Reveals the Inhibitory Mechanism of ICAT. *Mol Cell* 10, 563–571 (2002).

19. P. R. Elliott, *et al.*, Molecular Basis and Regulation of OTULIN-LUBAC Interaction. *Mol Cell* 54, 335–348 (2014).

20. N. R. Brown, M. E. M. Noble, J. A. Endicott, L. N. Johnson, The structural basis for specificity of substrate and recruitment peptides for cyclin-dependent kinases. *Nat Cell Biol* 1, 438–443 (1999).

21. Y. S. Chang, *et al.*, Stapled α−helical peptide drug development: A potent dual inhibitor of MDM2 and MDMX for p53-dependent cancer therapy. *Proc National Acad Sci* 110, E3445–E3454 (2013).

22. A. Peier, *et al.*, NanoClick: A High Throughput, Target-Agnostic Peptide Cell Permeability Assay. *Acs Chem Biol* 16, 293–309 (2021).

23. S. Aubry, *et al.*, Cell-surface thiols affect cell entry of disulfide-conjugated peptides. *Faseb J* 23, 2956–2967 (2009).

24. T. K. Sawyer, V. Guerlavais, K. Darlak, E. Feyfant, Macrocycles in Drug Discovery. *Drug Discov*, 339–366 (2015).
